# Potential of the C Genome of the Different Variants of *Brassica oleracea* for Heterosis in Spring *B. napus* Canola

**DOI:** 10.3389/fpls.2019.01691

**Published:** 2020-01-17

**Authors:** Azam Nikzad, Berisso Kebede, Jaime Pinzon, Jani Bhavikkumar, Xin Wang, Rong-Cai Yang, Habibur Rahman

**Affiliations:** ^1^ Department of Agricultural, Food and Nutritional Science, University of Alberta, Edmonton, AB, Canada; ^2^ Northern Forestry Centre, Natural Resources Canada, Edmonton, AB, Canada; ^3^ Crop Research and Extension Division, Alberta Agriculture and Rural Development, Edmonton, AB, Canada

**Keywords:** *Brassica napus*, *Brassica oleracea*, Heterosis, seed yield, canola quality traits

## Abstract

The genetic base of *Brassica napus* canola need to be broadened for exploitation of heterosis at a greater level in the breeding of F_1_ hybrid canola cultivars. In this study, we evaluated 228 inbred *B. napus* canola lines derived from six *B. napus* × *B. oleracea* interspecific crosses and following two breeding methods (F_2_- and BC_1_-derived lines) to understand the effect of the *B. oleracea* alleles on heterosis for different agronomic and seed quality traits. Test hybrids of the inbreds derived from crosses involving vars. *botrytis* (cauliflower), *alboglabra* (Chinese kale) and *capitata* (cabbage) cv. Badger Shipper, on an average, gave about 10% mid-parent heterosis (MPH), and about 67% of the test hybrids gave higher seed yield than the common *B. napus* parent indicating that *B. oleracea* alleles can contribute to heterosis for seed yield in spring *B. napus* canola hybrids. This was also evident from a positive correlation of the genetic distance of the inbred lines from the common *B. napus* parent with MPH for seed yield (*r* = 0.31) as well as with hybrid yield (*r* = 0.26). Almost no correlation was found between genetic distance and MPH for seed oil and protein content as well as with the performance of the test hybrids for these two traits. The occurrence of positive correlation between seed yield of the inbred lines and test hybrids suggested the importance of the genes exerting additive effect for high seed yield in the hybrids. Very little or almost no heterosis was found for the other agronomic traits as well as for seed oil and protein content. While comparing the two breeding methods, no significant difference was found for seed yield of the test hybrids or the level of MPH; however, the BC_1_-derived inbred and test hybrid populations flowered and matured earlier and had longer grain-filling period than the F_2_-derived population. Thus, the results suggested that the *B. oleracea* gene pool can be used in the breeding of spring *B. napus* canola to improve seed yield in hybrid cultivars.

## Introduction

*Brassica napus* L. (AACC, 2*n* = 38) canola, also known as oilseed rape, is one of the most important oilseed crops in the world. It is an amphidiploid species as a result of hybridization between *B. oleracea* (C genome, *n =* 9) and *B. rapa* (A genome, *n =* 10) (U, N, 1935). *B. napus* is a young agricultural crop species—originated about 7,500 years ago (Chalhoub et al., 2014). Spring, winter and semi-winter growth habit types exist in this crop species; however, genetic diversity within the germplasm of a given growth habit type is quite narrow (Bus et al., 2011). Currently, hybrid canola cultivars have taken the majority of the market share; for example, in Canada more than 95% of the canola acreage is captured by this type of cultivars (Morrison et al., 2016). To develop high yielding hybrid cultivars, presence of genetic diversity between the hybrid-parent lines is needed (for review, see Rahman, 2013). The narrow genetic base of the canola crop germplasm is one of the limitations for increasing seed yield in hybrid cultivars through exploitation of heterosis or hybrid vigor (reviewed in Zou et al., 2010); therefore, introgression of exotic heterotic alleles into *B. napus* canola is needed. The need of broadening the genetic base of *B. napus* canola through introgression of allelic diversity from *B. rapa* and *B. oleracea* has been suggested by several researchers (Jesske et al., 2013; Rahman, 2013; Zhang et al., 2015; for review, see Wang et al., 2017).

The association of hybrid vigour with genetic diversity as well as with general combining ability (GCA) of the parents has been investigated by different researchers in oilseed *B. napus*. Genetic divergence of the parental lines is thought to be related to the superior performance of the F_1_ hybrid (Sernyk and Stefansson, 1983; Lefort-Buson et al., 1986; Ali et al., 1995; Diers et al., 1996; Basunanda et al., 2007; Tan et al., 2007; Sang et al., 2015); however, this relationship has been found not to be always strong in this crop (Yu et al., 2005; Qian et al., 2007; Qian et al., 2009; Girke et al., 2012; Luo et al., 2016). GCA of the parents also seems to play an important role in this association (Diers et al., 1996; Qian et al., 2009; Tian et al., 2017).

Several studies have been conducted to evaluate the effect of the alleles of the primary and secondary gene pools of Brassica on heterosis in oilseed *B. napus*. For example, in case of the primary gene pool, Udall et al. (2004) found heterosis for seed yield in F_1_ hybrids developed by crossing of spring *B. napus* lines, carrying alleles introgressed from Chinese semi-winter type, with natural spring *B. napus*. Similarly, Quijada et al. (2004) found that introgression of alleles from winter *B. napus* can improve seed yield in spring *B. napus* canola hybrids. In case of the secondary gene pool, Qian et al. (2003) showed that Chinese *B. rapa* can be a valuable gene pool for alleles for increasing biomass yield in *B. napus*, and greater biomass at vegetative and maturity stage was found to be associated with higher seed yield in spring canola hybrids (Zhang and, Flottmann 2016). Genome contents introgressed from *B. rapa* into *B. napus* has also been found to improve seed yield in *B. napus* hybrids (Qian et al., 2005). Introgression of allelic diversity from *B. rapa* into Chinese semi-winter type, in fact, make this type genetically distinct from the European and Canadian spring *B. napus* (Qian et al., 2005; Qian et al., 2007), and alleles of the Chinese semi-winter type found to contribute to seed yield heterosis in spring or winter *B. napus* (Qian et al., 2007; Qian et al., 2009). However, very few studies have been so far conducted (Li et al., 2014; Rahman et al., 2015) to understand the effect of the *B. oleracea* alleles on heterosis in *B. napus* ‒ despite wide morphological and genetic diversity exist in this diploid species (Lázaro and Aguinagalde, 1998; Faltusová et al., 2011; Izzah et al., 2013; El-Esawi et al., 2016; Yousef et al., 2018). This suggests the need of understanding the *B. oleracea* gene pool for seed yield heterosis in *B. napus* canola hybrid. It has also been reported that genetic diversity in the C genome is lower than the A genome of *B. napus* (Bus et al., 2011; Delourme et al., 2013; Thakur et al., 2018); this enforce the need of increasing genetic diversity in the C genome of *B. napus* by introducing allelic diversity from *B. oleracea*—not only for increasing the level of heterosis in *B. napus* hybrid canola but also for continued improvement of the germplasm of this crop through breeding.

As reviewed above, wide diversity exists in *B. oleracea*; therefore, it can be hypothesized that the alleles of the different variants of *B. oleracea* would exhibit different levels of heterosis in *B. napus* hybrids. To our knowledge, no study has so far been conducted to understand the value of the different variants of *B. oleracea* for heterosis of agronomic and seed quality traits including seed yield in *B. napus* canola. In this study, we compared six *B. napus* canola populations, derived from *B. napus* × *B. oleracea* interspecific crosses involving a spring type *B. napus* canola line and six *B. oleracea* accessions belonging to four variants of the species for the level of heterosis in spring *B. napus* canola hybrids. Furthermore, the effect of limited backcrossing of the interspecific hybrid to the *B. napus* parent on the re-constituted *B. napus* canola lines for the level of heterosis has also been investigated.

## Materials and Methods

### Plant Materials

A total of 110 F_7_ lines derived from crossing of a single spring *B. napus* line A04-73NA to six *B. oleracea* accessions belonging to four variants of this species, viz. var. *alboglabra* line NRC-PBI, var. *botrytis* cv. BARI cauliflower, var. *capitata* cvs. Badger Shipper, Bindsachsener, and Balbro, and var. *italica* cv. Premium Crop, and 118 BC_1_F_6_ lines derived from crossing of the above-mentioned F_1_'s to the *B. napus* parent A04-73NA were used.

The spring *B. napus* parent A04-73NA is a canola quality line (zero erucic acid in oil and <15 µmol glucosinolate g^−1^ seed) developed by the Canola Program of the University of Alberta. All *B. oleracea* accessions were high-erucic (~40% erucic acid) and high glucosinolate (>60 µmol glucosinolate g^−1^ seed) type. Seeds of the *B. oleracea* accessions var. *alboglabra* line-NRC (PBI) was collected from the National Research Council, Saskatoon, Canada; var. *botrytis* cv. BARI cauliflower from the Bangladesh Agricultural Research Institute, Bangladesh; var. *italica* cv. Premium Crop from Dr. Ron Howard, Alberta Agriculture and Forestry, Brooks, Canada; var. *capitata* cv. Balbro from Hazera Seeds of Growth, Netherlands; and var. *capitata* cvs. Badger Shipper and Bindsachsener from germplasm collection of the Canola Program of the University of Alberta (Hasan et al., 2012). The interspecific crosses from where the above-mentioned inbred lines were developed are listed below (**Supplementary Table 1**):

- A04-73NA × *B. oleracea* var. *alboglabra* line NRC-PBI (Ol.alb.nrc, Chinese kale)
- A04-73NA × *B. oleracea* var. *botrytis* cv. BARI cauliflower (Ol.bot.cau, cauliflower)
- A04-73NA × *B*. *oleracea* var. *capitata* cv. Badger Shipper (Ol.cap.bad, cabbage)
- A04-73NA × *B. oleracea* var. *capitata* cv. Bindsachsener (Ol.cap.bin, cabbage)
- A04-73NA × *B. oleracea* var. *capitata* cv. Balbro (Ol.cap.bal, cabbage)
- A04-73NA × *B. oleracea* var. *italica* cv. Premium Crop (Ol.ita.pre, broccoli)

The F_1_ plants were self-pollinated to produce the F_2_ seeds and backcrossed to the *B. napus* parent to produce BC_1_ seeds. The F_2_ and BC_1_ population were subjected to pedigree breeding with selection for spring growth habit, plant fertility and the two canola quality traits, zero erucic acid in seed oil and low glucosinolate in seed meal. All 228 (110 + 118) inbred lines were confirmed to be spring type euploid *B. napus* (2*n* = 38) possessing the canola quality properties (Iftikhar et al., 2018). Theoretically, the C genome of this inbred population (A^n^A^n^C^n/o^C^n/o^) was expected to be composed of the C genome of *B. napus* (A^n^A^n^C^n^C^n^) and the C genome of *B. oleracea* (C°C°), where the proportion of *B. oleracea* alleles was expected to be 50% in case of the F_2_-derived population and 25% in case of the BC_1_-derived population.

### Production of Test Hybrids

Test hybrid seeds were produced by manual crossing of the 228 inbred lines as male and the B. napus line A04-73NA as female. For this, a total of 110 F7 plants of A04-73NA × B. oleracea and 118 BC1F6 plants of (A04-73NA × B. oleracea) × A04-73NA of the six crosses were grown in a greenhouse (21/18 ˚C ± 2˚ C day/night, 16 h photoperiod) of the Department of Agricultural, Food and Nutritional Science, University of Alberta in 2014–15 winter, and test hybrid seeds as well as self-pollinated F8 and BC1F7 generation seeds were produced for field trial in 2015. Two to three plants of each of the F8 and BC1F7 lines were grown in greenhouse in 2015-16 winter and their test hybrid seeds were produced for field trial in 2016. In the same way, test hybrid seeds were produced in greenhouse for 2017 and 2018 field trials.

### Field Trial

The test hybrids, their self-pollinated male parent lines, and the common *B. napus* parent A04-73NA were grown in field plots in the summer of 2015, 2016, 2017, and 2018. In 2015, the trial was grown at Edmonton Research Station (ERS), and in the remaining years, the trials were grown at St. Albert Research Station of the University of Alberta.

In 2015, seeding was done manually in 3-row plots of 1.0 × 1.2 m (length × width) size where 66 seeds were seeded in 22 spots in the middle row with 5 cm spacing between the spots, while 44 seeds were seeded in each of the two guard rows. After crop establishment (two leaf stage), thinning was done in the middle row where 22 ± 2 plants were retained. Plot size in 2016 was 2.0 m × 1.3 m (length × width) and in 2017 and 2018 was 3.0 m × 1.3 m; however, the number rows per plot in 2016 and 2017 was four while in 2018 it was two. In all these three years, seeding was done by a plot seeder. Amount seed used per plot was 1.5 g in 2016, and 2.0 g in 2017 and 2018. The difference in the size of the plots in different years was due to the availability of the test hybrid seeds.

Field plots were laid out in a way that the test hybrids were bordered by their respective parents, where the hybrid and its two parents constituted one experimental unit. This design enabled direct comparison of the test hybrids with their parents and precise estimation of mid-parent heterosis (MPH); however, this design had also increased the total number of plots in a replication to 513. To accommodate this large number of plots in a best uniform piece of a land, field plots were laid out in an incomplete randomized block design, where each replication was divided into multiple blocks. Number of replications in all year was two, and randomization of the experimental units (hybrid and parents) was done using Cropstat version 7.2 and Microsoft Excel 2007.

### Agronomic and Seed Quality Traits

The following agronomic and seed quality traits were collected from the middle rows on plot basis: Days to flowering, plant height (cm), days to maturity, seed yield (kg ha^−1^), and seed oil (%) and protein (%) contents. In addition to this, the duration of flowering time and grain-filling period data was collected in 2017 and 2018. Days to flowering data were collected when about 50% plants in a plot had at least one open flower. The end of flowering data was collected when about 90% plants in a plot did not have flower. Days to flowering and end of flowering data was collected two times in a week. Duration of flowering time was calculated by subtracting days to flower from the end of flowering date. Plant height (cm) data was collected at the end of flowering on a whole plot basis by measuring height of the plants from soil surface. Days to maturity data was collected from the middle rows when silique color changed from green to light yellow or brown and seed color of the silique on the main branch (examined by opening the silique) changed to brown or black. Grain-filling period was calculated by subtracting the end of flowering date from the maturity date. Plots were harvested with a plot combine and plot yield data was converted to kg/ha.

Seed oil and protein contents were estimated using near-infrared spectroscopy (NIRS, Model 6500, Foss North America, Eden Prairie, MN) in the Analytical Laboratory of the Canola Program of the University of Alberta. For this, five to eight gram bulk open-pollinated seeds harvested from the field plots were used. A calibration equation available in this laboratory was used for quantification of oil and protein contents using the software WinISI II (Infrasoft International, LLC.). Oil and protein contents were calculated on whole seed basis at 8.5% moisture and reported as percent of the whole seed.

### Simple Sequence Repeat (SSR) Marker Analysis

Genomic DNA of the above-mentioned 227 (110 + 117) F_2_- and BC_1_-derived inbred lines and their seven parents (*B. napus* A04-73NA and six *B. oleracea* cultivars/lines) was extracted using SIGMA DNA extraction kit (Sigma-Aldrich, St. Louis, MO, USA) following manufacture's instruction. A total of 95 polymorphic SSR markers (Nikzad et al., 2019) from nine C-genome linkage groups (LG) were selected from 418 markers for genotyping the populations. Polymerase chain reactions (PCR) for amplification of the genomic DNA was performed in a total volume of 15.5 µl, which included 20 ng genomic DNA, 5× PCR reaction buffer, 25 mM MgCl_2_, 0.6 unit *Taq* DNA polymerase (Promega Corporation, Madison, WI), 10 mM each dNTP (Invitrogene Life Technologies Inc., Burlington, ON), 5 µM of each forward and reverse primer, and 5 µM tag F (fluorescent dyes FAM, VIC, NED, and PET). PCR was carried out in a GeneAmp PCR System 9700 thermal cycler (Applied Biosystems, Foster City, CA) with the following program: 1 cycle of 5 min at 95°C for initial denaturation, 35 cycles where each cycle consisted of 1 min at 95°C for denaturation, 1 min at 58°C for annealing and 1.5 min at 72°C for extension, and the final extension time was 15 min at 72°C. Size-based separation of the amplified DNA fragments was done using a capillary electrophoresis AB Genetic Analyzer No. 3730 (Applied Biosystems, Foster City, CA).

### Statistical Analysis

Best linear unbiased prediction (BLUP) was used to estimate genotypic value of the inbred lines and their test hybrids for different agronomic and seed quality traits. In this analysis, replication, block nested in replication and genotype were considered as random effects, and the analysis was done using the statistical software program of SAS (SAS Institute, 2010).

***Heterosis***: MPH (mid-parent heterosis) and NPH (heterosis over the *B. napus* parent) was calculated for different agronomic and seed quality traits using the estimates from BLUP and using the following formulas:

$$\text{Mid-parent value}=\frac{\text{Parent}\;1+\text{Parent}\;2}{2}\;$$

$$\text{MPH}=\frac{\text{Test hybrid value }-\text{ Mid-parent value}}{\text{Mid-parent value}}\;\times \;100$$

$$\text{NPH}=\frac{\text{Test hybrid }v\text{alue}-\text{A}04-73\text{NA value}}{\text{A}04-73\text{NA value}}\;\times 100$$

***Analysis of variance (ANOVA)***: ANOVA for different traits was performed using data of the inbred lines and test hybrids, and MPH and NPH values with the statistical software program of SAS using PROC MIXED through the option METHOD = Type 3 sums of squares. In this analysis, environment consisted of four field trials conducted over four years (2015, 2016, 2017 and 2018) as detailed in the *Field Trial* section. The inbred lines used in this study to produce the test hybrids were developed from six crosses (Ol.alb.nrc, Ol.bot.cau, Ol.cap.bad, Ol.cap.bin, Ol.cap.bal and Ol.ita.pre) and following two breeding methods (F_2_- and BC_1_-derived). The six crosses, the two breeding methods and their interaction (cross × method) were considered as fixed effect; while the environment and genotype nested in cross × method were considered as random effect. The following linear model summarizes the sources of variation:

$$Y_{ijkp\;=\;}\mu \;+\;C_{i}+\;T_{j}+\;{\left(CT\right)}_{ij}+\;E_{k}\;+\;G_{p(ij)}\;+\;GE_{kp(ij)}\;+\;\epsilon_{ijkp}$$

where *Y_ijkp_* is the trait value observed for the *p*th genotype (inbred line) from the *i*th (*i =* 1, …, 6) cross and the *j*th (*j =* 1, 2) breeding method grown in the *k*th (*k =* 1, …, 4) environment; *µ*, *C_i_*, *T_j_,* (*CT*)*_ij_*, *E_k_*, *G_p(ij)_* and *GE_kp(ij)_* are the overall mean and effects due to the *i*th cross, the *j*th breeding method, the *ij*th cross × breeding method, the *k*th environment, the *p*th genotype within the *ij*th cross × breeding method and the *kp*th genotype × environment interaction, respectively; *ϵ_ijk__p_* is the random residual for the *ijkp*th observation. All random effects (*E_k_*, *G_p(ij)_* and *GE_kp(ij)_*) and random residual are assumed to be independently and identically distributed with mean zero and variances being $\sigma_{E}^{2}$, $\sigma_{G}^{2}$, $\sigma_{GE}^{2}$ and $\sigma_{\epsilon }^{2}$, respectively, i.e., *E_k_* ~ *N*(0, $\sigma_{E}^{2}$); *G_p(ij)_* ~ *N*(0, $\sigma_{G}^{2}$) and *G_kp(ij)_* ~ *N*(0, $\sigma_{GE}^{2}$) and *ϵ_ijk__p_* ~ *N*(0, $\sigma_{\epsilon }^{2}$).

***Least square means*:** LSmeans of the fixed effects were calculated with SAS based on the estimates from BLUP, and test for significant difference (α ≤ 0.05) between the Lsmeans was done following Tukey's test. Pearson's correlation coefficient (*r*) values were calculated using cor.test function and chart.correlation of the PerformanceAnalytics package (Peterson et al., 2015).

***Multivariate analyses*:** Principal component analysis (PCA) was used to facilitate the identification of the test hybrids with differentiated performance when accounting for differences and interrelationships among the multiple agronomic and seed quality traits. Lsmean values of the test hybrids (obtained above) across the environments for different agronomic and seed quality traits were used for the PCA and data standardized (mean = 0, variance = 1) prior to this analysis. Data standardization and PCA were performed using R package vegan (Oksanen et al., 2019) according to Borcard et al. (2018).

***Genetic diversity analysis*:** The fragment analysis results from ABI were scored for presence or absence of marker alleles using the software program GeneMarker^®^ version 2.4.0 (SoftGenetics LLC, State College, PA); however, all genotyping results were confirmed manually as well. The absence (0) or presence (1) of the amplification products were scored based on fragment length, and data recorded in a 0/1 matrix for the presence/absence of the marker amplicons. The 0/1 matrix was used to calculate Nei's genetic distance using the software GENALEX 6 (Peakall and Smouse, 2006), and Pearson's correlation (*r*) between genetic distance of the inbred lines from the common *B. napus* parent and performance of the inbred lines and test hybrids was calculated for different agronomic and seed quality traits using cor.test function and chart.correlation in R statistical computing program (Peterson et al., 2015).

## Results

### Agronomic and Seed Quality Traits

**Figure1:**
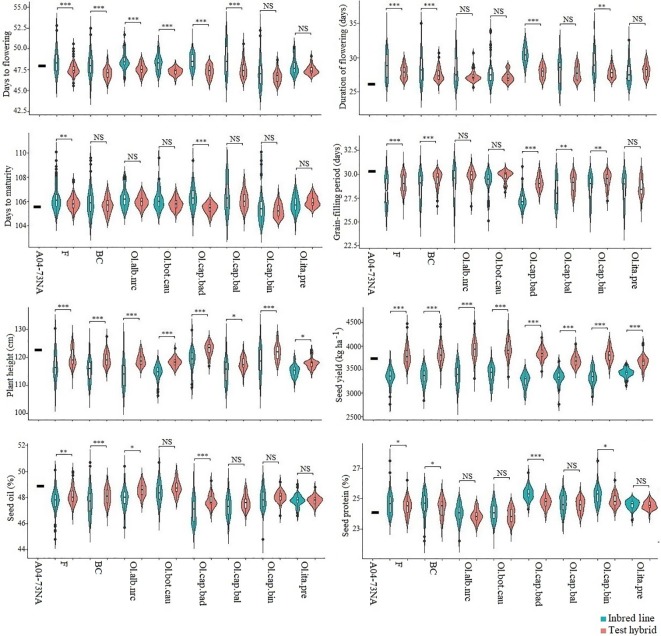
Violin plot of six inbred populations derived from six *Brassica napus* × *B. oleracea* interspecific crosses and following two breeding methods (F_2_- and BC_1_-derived) and their test hybrids. Data of the common *B. napus* parent A04-73NA is also included. Teal bars represent inbred lines and blush bars represent the test hybrids. Ol.alb.nrc = *B. napus* (A04-73NA) × *B. oleracea* var. *alboglabra* line NRC-PBI (*n* = 36); Ol.bot.cau = *B. napus* (A04-73NA) × *B. oleracea* var. *botrytis* cv. BARI cauliflower-1 (*n* = 40); Ol.cap.bad = *B. napus* (A04-73NA) × *B. oleracea* var. *capitata* cv. Badger Shipper (*n* = 33); Ol.cap.bal = *B. napus* (A04-73NA) × *B. oleracea* var. *capitata* cv. Balbro (*n* = 42); Ol.cap.bin = *B. napus* (A04-73NA) × *B. oleracea* var. *capitata* cv. Bindsachsener (*n* = 43); Ol.ita.pre = A04-73NA × *B. oleracea* var. *italica* cv. Premium Crop (*n* = 34) F = F_2_-derived population (*n* = 110); BC = BC_1_ (F_1_ × *B. napus*)-derived population (*n* = 118). *, **, *** indicate significant at *P* < 0.05, < 0.01, and < 0.001, respectively; NS indicate not statistically significant.

**Figure 2:**
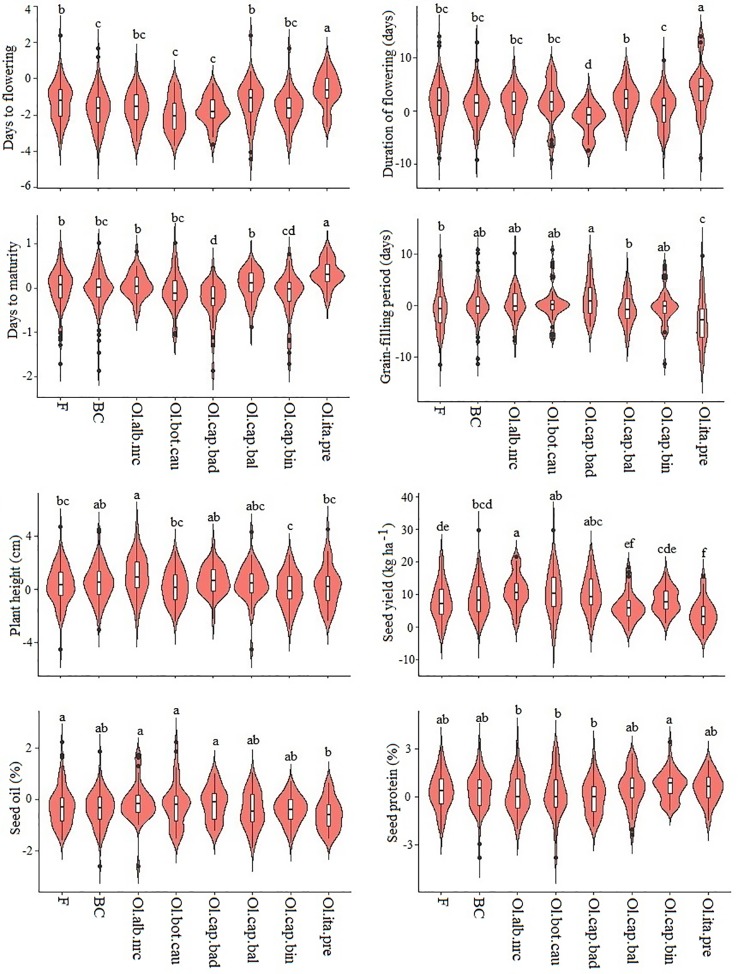
Violin plot of six test hybrid populations developed using six inbred populations derived from six *Brassica napus* × *B. oleracea* interspecific crosses and following two breeding methods (F_2_- and BC_1_-derived) for mid-parent heterosis (MPH).Ol.alb.nrc = *B. napus* (A04-73NA) × *B. oleracea* var. *alboglabra* line NRC-PBI (*n* = 36); Ol.bot.cau = *B. napus* (A04-73NA) × *B. oleracea* var. *botrytis* cv. BARI cauliflower-1 (*n* = 40); Ol.cap.bad = *B. napus* (A04-73NA) × *B. oleracea* var. *capitata* cv. Badger Shipper (*n* = 33); Ol.cap.bal = *B. napus* (A04-73NA) × *B. oleracea* var. *capitata* cv. Balbro (*n* = 42); Ol.cap.bin = *B. napus* (A04-73NA) × *B. oleracea* var. *capitata* cv. Bindsachsener (*n* = 43); Ol.ita.pre = A04-73NA × *B. oleracea* var. *italica* cv. Premium Crop (*n* = 34). F = F_2_-derived population (*n* = 110); BC = BC_1_ (F_1_ × *B. napus*)-derived population (*n* = 118). LSmean values of the violin plots with the same letter are not significantly different at *P* < 0.05.

Among the six inbred populations, populations derived from the crosses involving broccoli gave the greatest seed yield, while the population derived from the cross involving cabbage cv. Badger Shipper gave the lowest yield (**Figure 1**; **Supplementary Table 2**); seed yield of the population derived from the cross involving cauliflower was comparable to the population derived from the cross involving broccoli. Test hybrid populations gave significantly (*P <*0.001) higher yield than their corresponding inbred populations (**Figure 1**; **Supplementary Figure 1**; **Supplementary Table 2**). Among the different populations, test hybrids of the populations derived from the crosses involving cauliflower, Chinese kale and two of cabbages cvs. Badger Shipper and Bindsachsener gave higher yield than the population derived from the crosses involving the other *B. oleracea* accessions including broccoli as well as the common *B. napus* parent A04-73NA. About 4.0 to 11.1% MPH was found for yield in the six populations—the highest MPH was found in the population derived from the cross involving Chinese kale and the lowest in the population derived from the cross involving broccoli (**Figure 2**; **Supplementary Tables 2** and **4**). About 2.5 fold greater magnitude of heterosis was observed in the test hybrid population derived from the cross involving Chinese kale (11.1 ± 2.2) as compared to test hybrid population derived from the cross involving broccoli (4.0 ± 2.2) (**Supplementary Table 2**). Wide variation was found for MPH within the test hybrid populations where individual hybrid producing up to 82.7% MPH was identified (data not shown). In all cases, MPH was higher than NPH (**Supplementary Table 2**). The highest NPH for seed yield was observed in the population derived from the cross involving Chinese kale (4.8%) while the lowest in the population derived from the cross involving broccoli (0.2%) (**Figure 2**); individual test hybrid exhibiting with up to 63.8% NPH was identified within the population derived from the cross involving cauliflower (data not shown). While comparing the two breeding methods (F_2_- and BC_1_-derived) for seed yield, no significant difference was found between these methods for the development of the inbred lines as well as in their test hybrid populations (**Figure 1**; **Supplementary Table 5**). Average MPH of the BC_1_-derived population was 8.8% while it was 8.0% for the F_2_-derived population (**Figure 2**; **Supplementary Table 3**).

As compared to seed yield, much less contrasting difference was found between the inbred and test hybrid (**Figure 1**) and between MPH and NPH (**Figure 2**) for days to flowering and maturity. In general, the test hybrid populations flowered significantly (*P <* 0.001) earlier, had shorter duration of flowering and took longer grain-filling period than the inbred populations—these factors might have contributed to the greater seed yield in the test hybrid populations (**Figure 1**; **Supplementary Tables 2** and **3**). MPH for these flowering and maturity traits was very low—in most cases less than 2.0% (**Figure 2**; **Supplementary Table 2**).

While comparing the populations developed following two breeding methods, the BC_1_-derived inbred population flowered (47.9 ± 0.67) and matured (106.0 ± 1.3) significantly (*P <* 0.05) earlier than the F_2_-derived population. Test hybrid populations of the BC_1_-derived lines still flowered earlier (47.1 ± 0.6, *P <* 0.05) and had longer grain-filling period (29.5 ± 5.4) than test hybrid population of the F_2_-derived lines (**Supplementary Tables 3** and **5**).

In contrast to the above-mentioned flowering and maturity traits, test hybrid populations of all crosses were significantly (*P <* 0.05) taller than the inbred populations (**Figure 1**) and exhibited significantly greater MPH than NPH (**Figure 2**); however, the extent of MPH (1.0%) and NPH (−1.0 to −2.8%) was negligible (**Supplementary Table 2**).

Among the six populations, inbred and test hybrids of the crosses involving cauliflower had the greatest seed oil content (**Figure 1**; **Supplementary Table 2**). Almost no MPH was found for seed oil (−0.1 to −0.6%) and protein (0.0 to 0.7%) contents, suggesting the importance of additive effect of the genes in the genetic control of these two seed quality traits (**Supplementary Table 2**).

### Correlation

**Figure 3:**
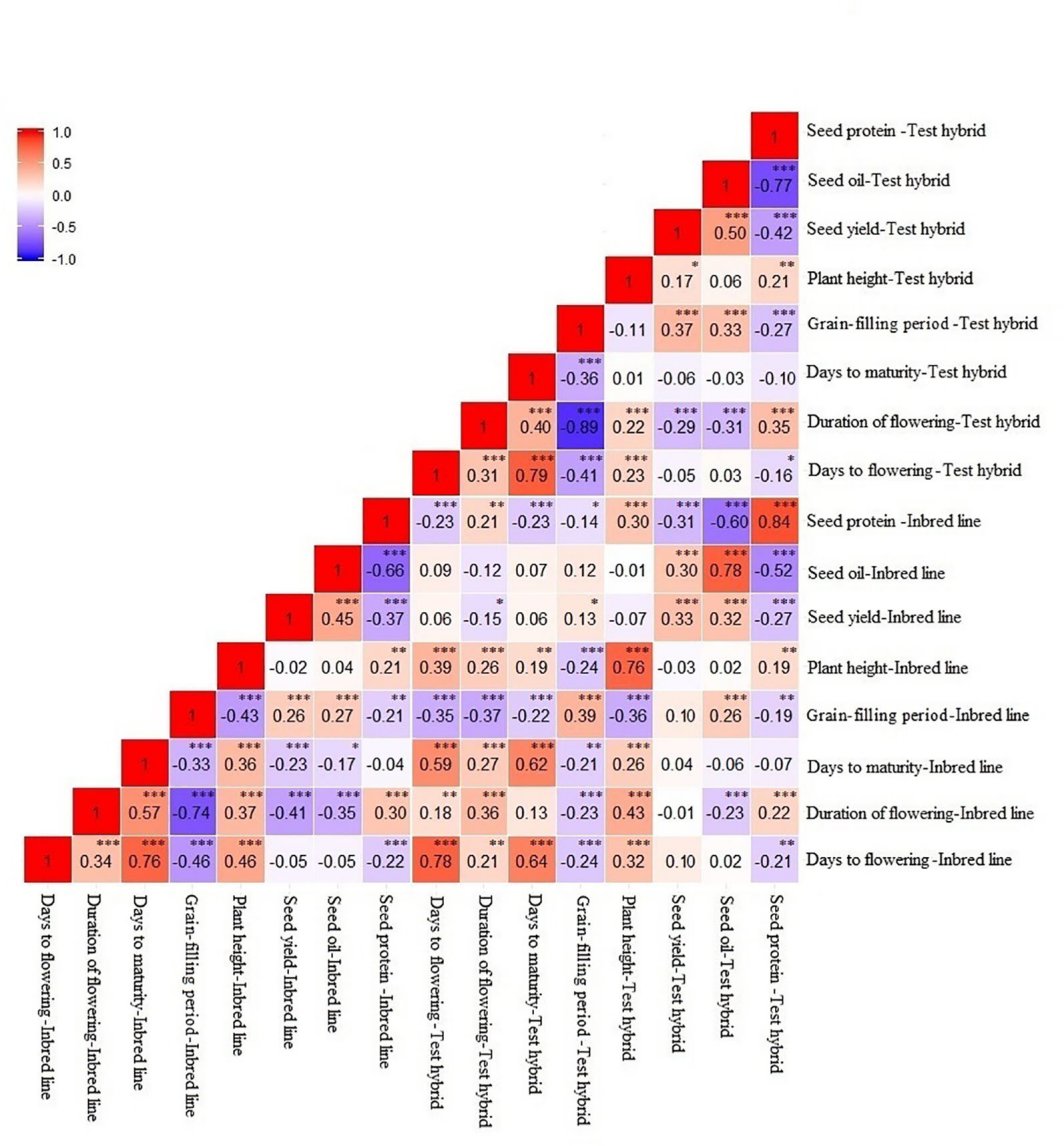
Correlation between different agronomic and seed quality traits in an inbred population of 228 lines derived from six *Brassica napus* × *B. oleracea* interspecific crosses and in their test hybrids. The strength and direction of the correlation are indicated by the color: Red represents the positive correlation while blue represents the negative correlation; the intensity of the color indicates the strength of the correlation. *, **, *** indicate significant at *P* < 0.05, < 0.01, and < 0.001, respectively; NS indicate not statistically significant.

Days to flowering showed a positive correlation (*P <* 0.001) with duration of flowering and days to maturity, while it showed a negative correlation (*P <* 0.001) with grain-filling period in both inbred and test hybrid populations (**Figure 3**). No significant correlation of this trait was found with seed yield; however, seed yield showed a significant (*P <* 0.001) negative correlation with duration of flowering, and a positive correlation with grain-filling period and seed oil content in both inbred and test hybrid populations. This suggests that high yielding lines or test hybrids with high oil content and earliness of flowering and maturity and longer grain-filling period can be obtained from this population.

**Figure 4:**
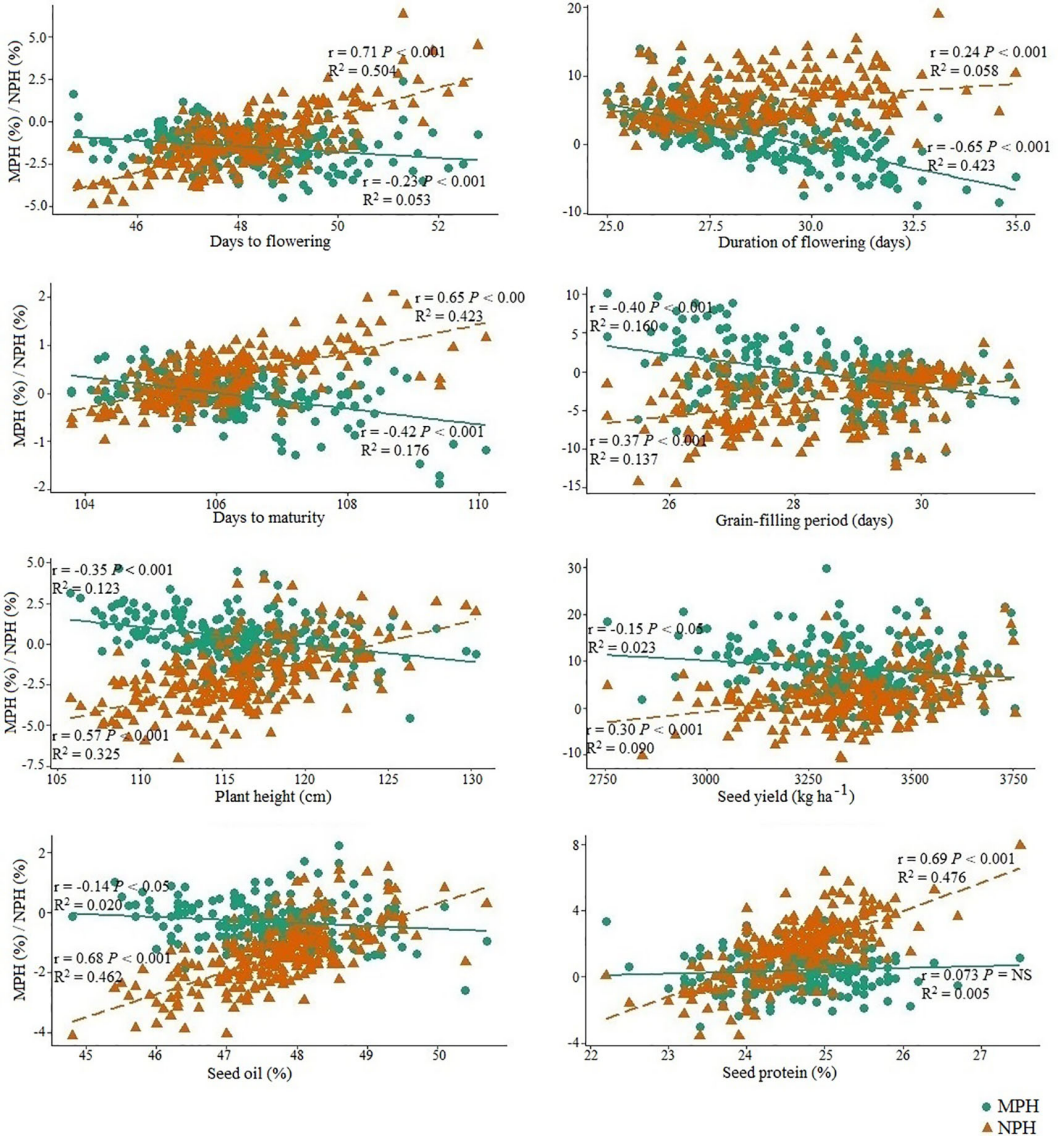
The relationship of the performance of the inbred lines (*n =* 228) derived from six *Brassica napus* × *B. oleracea* interspecific crosses with mid-parent heterosis (MPH) and heterosis over the common *B. napus* parent (NPH) for different agronomic and seed quality traits. Green circles and green solid lines represent MPH and orange triangles and orange broken line represents NPH.

Correlation between the performance of inbred lines and their test hybrids was studied to investigate the extent of the effect of the inbred lines on the performance of the hybrids for different agronomic and seed quality traits including seed yield. The inbred population showed significant positive correlation (*P <* 0.001) with the test hybrid population for all agronomic and seed quality traits (**Figure 3**). This suggests that additive genes play an important role in the genetic control of these traits; therefore, improvement of these traits in the inbred lines will be needed for the improvement of these traits in the hybrids. However, in the cases of seed yield, duration of flowering and grain-filling period, the *r* values of ≤0.40 suggests that significant amount of non-additive effect of the genes are also involved in the genetic control of these traits. For majority of the traits, performance of the inbred lines showed significant (*P <* 0.05) negative correlation with MPH; however, correlation between the performance of the inbred lines and NPH was significant (*P <* 0.001) and positive for all traits, including seed yield (*r* = 0.30) (**Figure 4**).

### Genetic Distance and Molecular Marker Analysis

**Figure 5:**
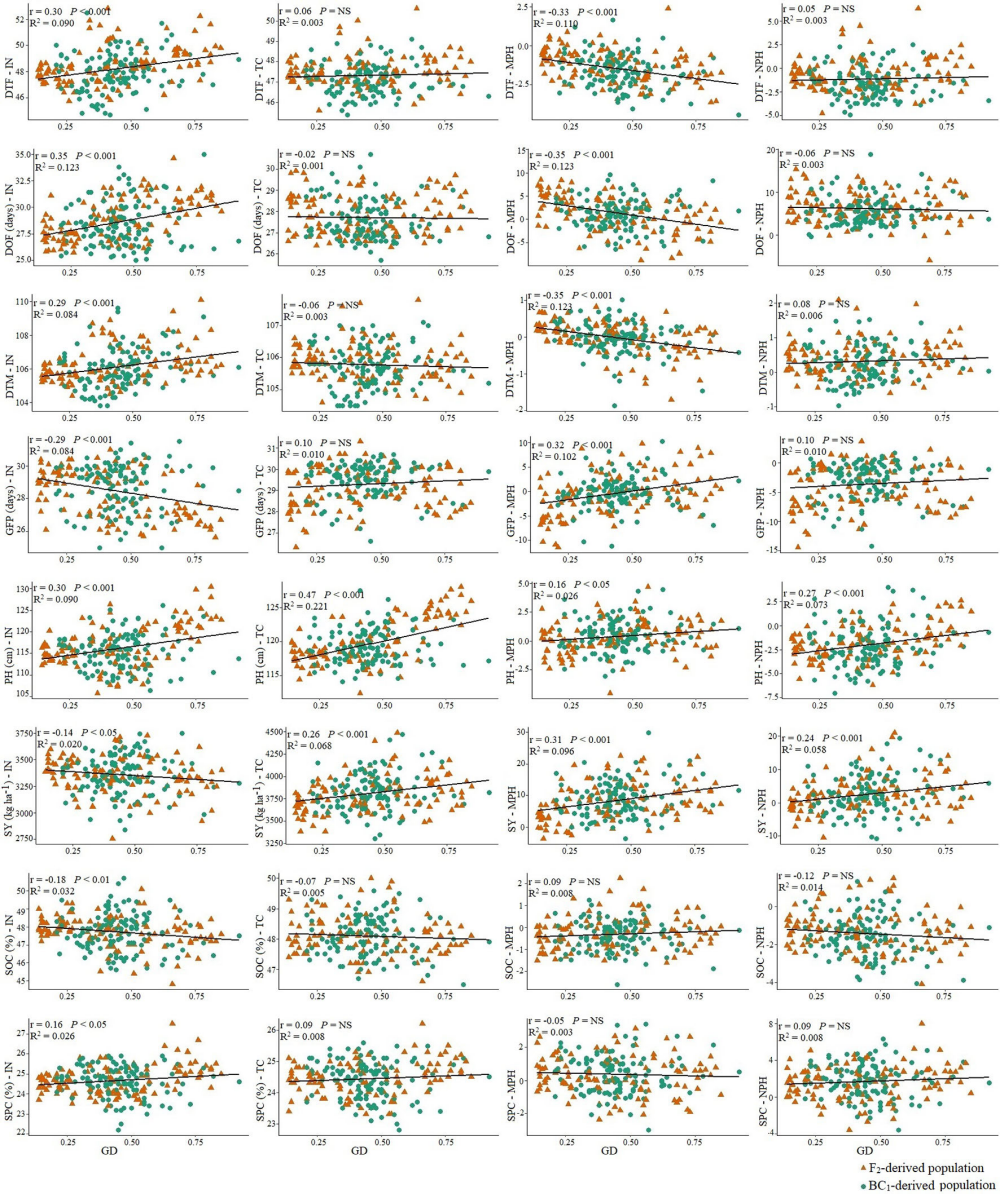
The relationship of the genetic distance (GD) of the inbred lines (*n* = 228), derived from six *Brassica napus* × *B. oleracea* interspecific crosses and developed following two breeding methods (F_2_- and BC_1_-derived populations), with the performance of the inbred lines (IN), their test hybrids (TC), and with mid-parent heterosis (MPH) and heterosis over the common *B. napus* parent (NPH) for different agronomic and seed quality traits. Orange dots represent the F_2_-derived population and green dots represents the BC_1_-derived population DTF, Days to flowering; DOF, Duration of flowering; DTM, Days to maturity; GFP, Grain-filling period; PH, Plant height; SY, Seed yield; SOC, Seed oil content; SPC, Seed protein content.

To understand the effect of genetic distance of the inbred lines from the *B. napus* parent on the level of heterosis in test hybrids, correlation between genetic distance and the performance of inbred lines, test hybrids as well as MPH and NPH was calculated. Genetic distance of the inbred lines from the *B. napus* parent showed a weak negative correlation (*r* = −0.14) with seed yield in the inbred population; however, this correlation was positive (*r* = 0.26) in the test hybrid population as well as with MPH (*r* = 0.31) and NPH (*r* = 0.24) (**Figure 5**). A moderate to weak positive correlation of genetic distance was found with days to flowering (*r =* 0.30), duration of flowering (*r =* 0.35), and days to maturity (*r =* 0.29) in the inbred population; however, this correlation was negligible in the test hybrid population. A positive correlation of the genetic distance of the inbred lines with days to flowering, duration of flowering and days to maturity indicate that *B. oleracea* alleles delayed flowering and maturity in the inbred lines; however, the negative effect of these *B. oleracea* alleles has been repressed to some extent by the alleles of *B. napus* in the test hybrids. Genetic distance showed a negative correlation (*r =* −0.29) with grain-filling period in the inbred population; however, this correlation was positive (*r* = 0.32) for MPH. A moderate positive correlation of genetic distance was found with plant height in the inbred (*r =* 0.30) and test hybrid population (*r =* 0.47) as well as for MPH and NPH. Almost no correlation was found between genetic distance and seed oil or protein content in both inbred and hybrid population (**Figure 5**).

It was expected that the test hybrid population to be heterozygous at different loci for the alleles originating from the *B. oleracea* and *B. napus* parents. The position of the 95 SSR markers used in this study together with heterozygosity of the markers in the test hybrid population, deduced from marker genotype of the inbred lines and the common *B. napus* parent A04-73NA, is presented in **Supplementary Figure 2**. Of the 95 SSR markers, heterozygous loci could be deduced in the test hybrid population for 89 (93.7%) markers. For a given marker, the proportion (%) of loci to be heterozygous in the test hybrid population varied from 0.5 to 55.4% with being only six markers showing heterozygosity 78.6 to 100%; the average heterozygosity of the 89 markers in the entire population was 19.6%. Among the different chromosomes, markers from the chromosome C7 (28.9%) showed the greatest and the markers from C9 (12.1%) showed the least heterozygosity.

### Performance of the Top, Medium and Poor Inbred Lines in Test Hybrids

**Figure 6:**
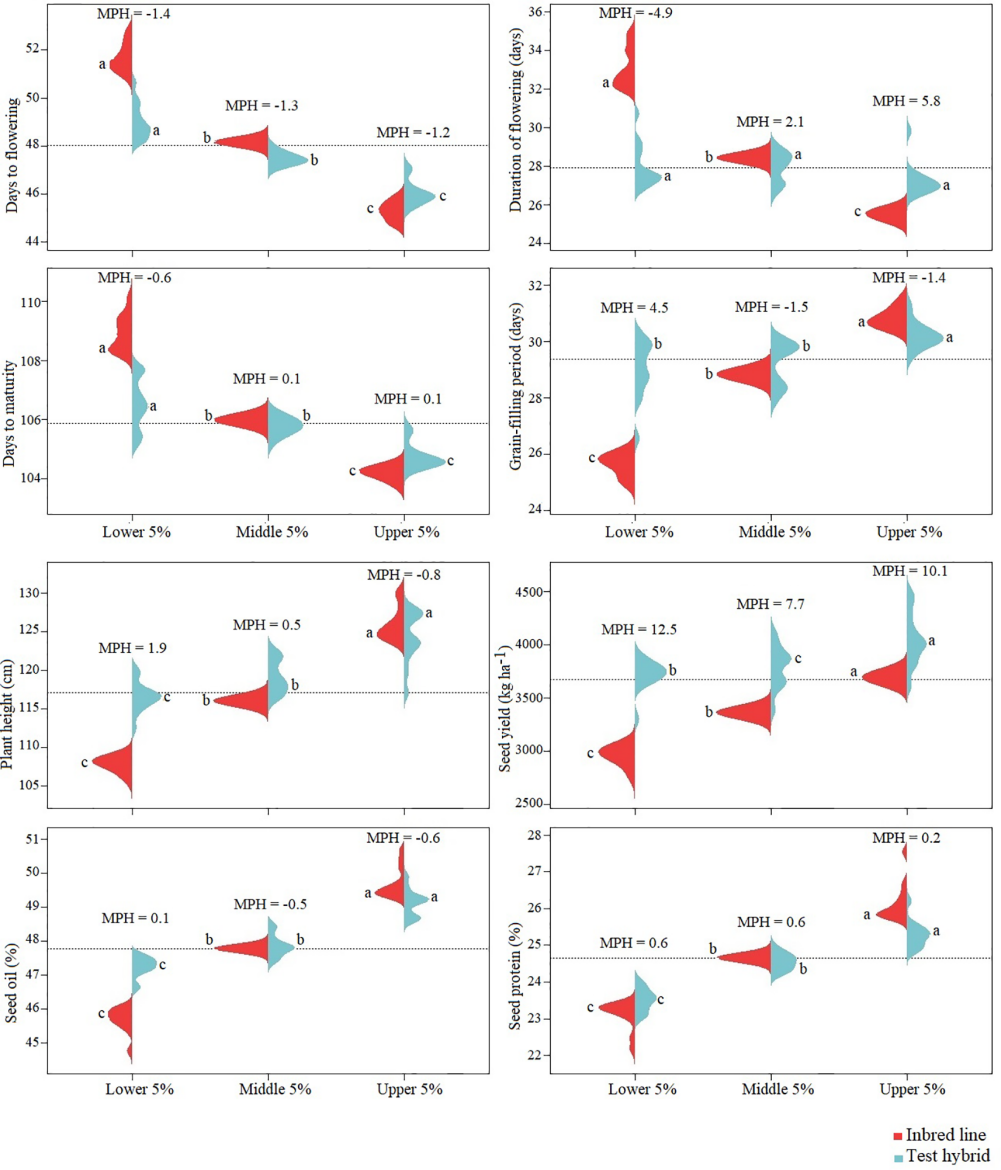
Beanplot of the 5% top, medium and poorest performing inbred lines, derived from *Brassica napus* × *B. oleracea* interspecific crosses, and the performance of their test hybrids. Red color represents the inbred lines and blue color represents the test hybrids. MPH = percent mid-parent heterosis. Inbred lines and test hybrids are compared separately; the same letter for the inbred or hybrid indicates the values are not significantly different at *P <* 0.05.

The performance of the top 5%, poorest 5% and 5% medium yielding inbred lines were compared with their hybrids as well as for the level of MPH. Among these three groups, greatest MPH was found in the hybrids of the poorest inbred lines. However, test hybrids of the top 5% inbred lines gave significantly greater seed yield than test hybrids of the other two groups indicating the importance of both additive and non-additive genes of the hybrid parents for increased seed yield in F_1_ hybrids (**Figure 6**).

In case of the other agronomic and seed quality traits, the top performing inbred lines also resulted the best performing hybrids indicating the importance of the additive effect genes in the control of these traits. Among these traits, least difference between the performance of the hybrids of the top and poorest inbred lines was found for duration of flowering and grain-filling period indicating the importance of non-additive effect of the genes in the genetic control of these traits; this was also evident from the occurrence of about 5% MPH for these traits.

### Multivariate Analysis

**Figure 7:**
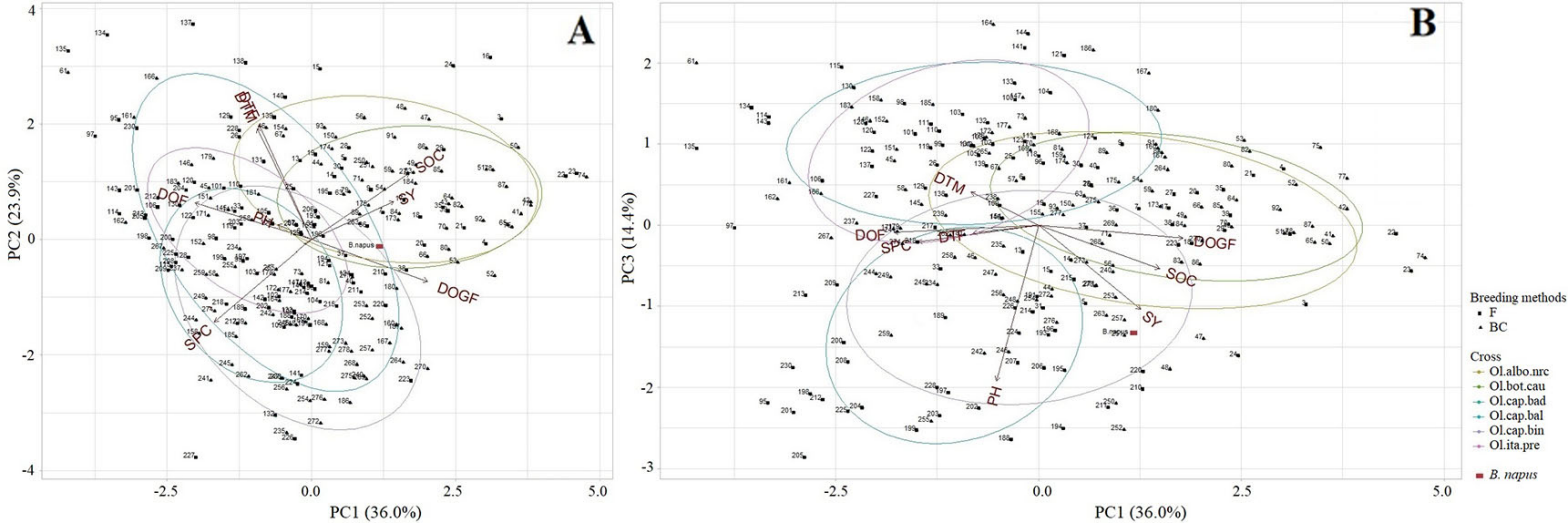
Principal component analysis biplot of the test hybrids (*n* = 228) of the inbred lines, derived from six *Brassica napus* × *B. oleracea* interspecific crosses and following two breeding methods (F_2_- and BC_1_-derived), and the common *B. napus* parent A04-73NA, illustrating the distribution of the test hybrids characterized by different agronomic and seed quality traits in the space of the first principal component (PC1) versus PC2 **(A)**, and PC1 versus PC3 **(B)**. The name of the inbred lines of the test hybrids are shown in **Supplementary Table 1**. DTF, Days to flowering; DOF, Duration of flowering; DTM, Days to maturity; DOGF, Duration of grain-filling period; PH, Plant height; SY, Seed yield; SOC, Seed oil content; SPC, Seed protein content; Ol.alb.nrc (mustard color) = *B. napus* (A04-73NA) × *B. oleracea* var. *alboglabra* line NRC-PBI (*n* = 36); Ol.bot.cau (green) = *B. napus* (A04-73NA) × *B. oleracea* var. *botrytis* cv. BARI cauliflower-1 (*n* = 40); Ol.cap.bad (teal) = *B. napus* (A04-73NA) × *B. oleracea* var. *capitata* cv. Badger Shipper (*n* = 33); Ol.cap.bal (blue) = *B. napus* (A04-73NA) × *B. oleracea* var. *capitata* cv. Balbro (*n* = 42); Ol.cap.bin (violet) = *B. napus* (A04-73NA) × *B. oleracea* var. *capitata* cv. Bindsachsener (*n* = 43); Ol.ita.pre (pink) = A04-73NA × *B. oleracea* var. *italica* cv. Premium Crop (*n* = 34); *B. napus* parent (red rectangle); F (solid square) = F_2_-derived population (*n* = 110); BC (solid triangle) = BC_1_ (F_1_ × *B. napus*)-derived population (*n* = 118).

***Test hybrid populations***: The first three PC explained 74.3% of the total variation (PC1: 36.0%, PC2: 23.9%, PC3: 14.5%) for different agronomic and seed quality traits. PC1 showed high correlation with various traits (**Supplementary Table 6**). The test hybrids with short duration of flowering but having long grain-filling period, and the test hybrids with high seed yield and high oil but low protein content were grouped together on right half of the plot (**Figure 7**), and were mostly derived from the crosses with Chinese kale and cauliflower. PC2 explained mostly a gradient of days to flowering and maturity, and seed protein content (**Supplementary Table 6**). The early flowering and maturing test hybrids, and the test hybrids with high seed protein content were grouped together on the lower half of the ordination plot (**Figure 7A**). The upper right part of the biplot (**Figure 7A**) showed that seed yield, seed oil content and grain-filling period were positively correlated and these three variables were negatively correlated with duration of flowering and seed protein content. A strong positive correlation between days to flowering and maturity was also reflected from close association of the vectors for these traits at the upper left part of the biplot (**Figure 7A**). PC3 explained a gradient of plant height and seed yield (**Supplementary Table 6**, **Figure 7B**). High yielding test hybrids with long stature, particularly those derived from the crosses with cabbages cvs. Badger Shipper and Bindsachsener, grouped together on the lower half of the ordination plot compared to the test hybrids derived from the crosses involving cabbage cv. Balbro and broccoli which tended to be distributed at the upper side of the biplot.

**Figure 8:**
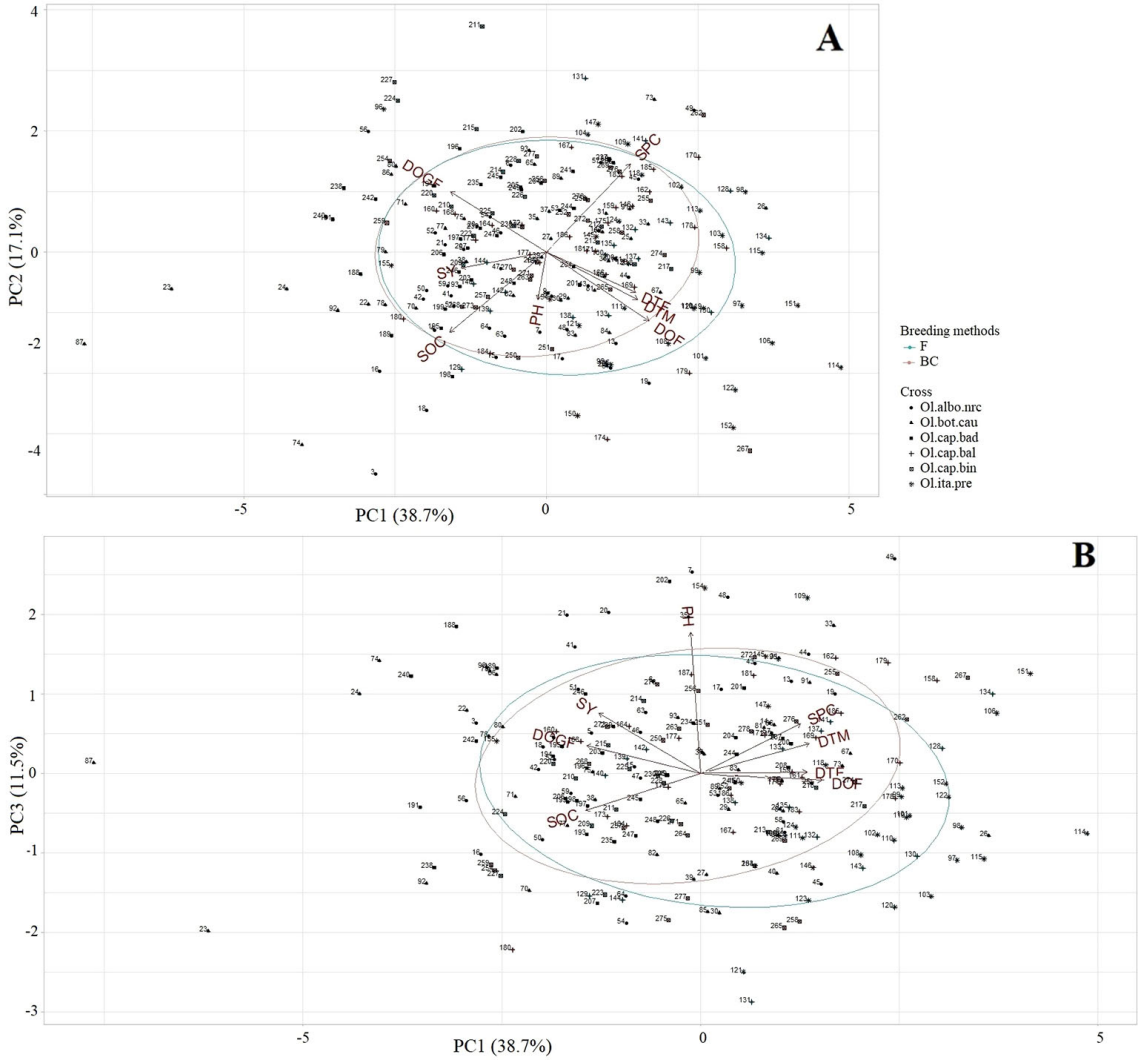
Principal component analysis biplot of mid-parent heterosis (MPH; *n* = 228) of the test hybrids of the inbred lines, derived from six *Brassica napus* × *B. oleracea* interspecific crosses and following two breeding methods (F_2_- and BC_1_-derived), illustrating the distribution of test hybrids characterized by different agronomic and seed quality traits in the space of the first principal component (PC1) versus PC2 **(A)**, and PC1 versus PC3 **(B)**. The name of the inbred lines of the test hybrids exhibiting mid-parent heterosis (MPH) are shown in **Supplementary Table 1**. DTF = Days to flowering; DOF = Duration of flowering; DTM = Days to maturity; DOGF, Duration of grain-filling period; PH, Plant height; SY, Seed yield; SOC, Seed oil content; SPC, Seed protein content; Ol.alb.nrc (soild circle) = *B. napus* (A04-73NA) × *B. oleracea* var. *alboglabra* line NRC-PBI (*n* = 36); Ol.bot.cau (solid triangle) = *B. napus* (A04-73NA) × *B. oleracea* var. *botrytis* cv. BARI cauliflower-1 (*n* = 40); Ol.cap.bad (solid square) = *B. napus* (A04-73NA) × *B. oleracea* var. *capitata* cv. Badger Shipper (*n* = 33); Ol.cap.bal (plus sign) = *B. napus* (A04-73NA) × *B. oleracea* var. *capitata* cv. Balbro (*n* = 42); Ol.cap.bin (empty square) = *B. napus* (A04-73NA) × *B. oleracea* var. *capitata* cv. Bindsachsener (*n* = 43); Ol.ita.pre (star) = A04-73NA × *B. oleracea* var. *italica* cv. Premium Crop (*n* = 34); *B. napus* parent (red rectangle); F (green circle) = F_2_-derived population (*n* = 110); BC (brown circle) = BC_1_ (F_1_ × *B. napus*)-derived population (*n* = 118).

***Mid-Parent heterosis*:** In case of MPH, the first three PC explained 67.4% of the total variation (PC1: 38.7%, PC2: 17.2%, PC3: 11.5%) for the agronomic and seed quality traits. PC1 showed high correlation with all traits except plant height (**Supplementary Table 6**). PC2 explained mostly a gradient of the duration of flowering, and seed oil and protein contents; whereas, PC3 explained a gradient of plant height (**Supplementary Table 6**). Individuals showing high MPH for seed yield, oil, protein, grain-filling period, and early flowering and maturity grouped together on the left half of the plot (**Figure 8A**), while, individuals showing high MPH for seed protein content grouped together on the upper right half of the ordination plot (**Figure 8A**). Plant height was more important trait for distribution of the individuals along the PC3 (**Figure 8B**); therefore, individuals showing high MPH for this trait grouped together on the upper half of the ordination plot (**Figure 8B**). No striking differences were observed between the F_2_- and BC_1_-derived individuals for most of the traits as was found based on LSmeans data (**Figure 8**).

## Discussion

Since identification of the phenomenon heterosis or hybrid vigour in maize, the development of F_1_ hybrid cultivars has received much attention to the breeders. Some field crops, such as maize, sunflower and canola, and vegetable crops, such as cabbage and cauliflower grown today are predominantly hybrid cultivars. Currently, hybrid cultivars of *B. napus* canola captured more than 90 percent of the total canola planted area in Canada (Morrison et al., 2016). However, the narrow genetic base of *B. napus* resulted from intensive selection by breeders is one of the bottlenecks for continual improvement of this type of cultivars for seed yield and other agronomic and seed quality traits (Jesske et al., 2013; Rahman et al., 2016; Zhao et al., 2016). Therefore, broadening the genetic base of the spring *B. napus* canola, especially its C genome which genetic base is known to be narrow as compared to its A genome (Bus et al., 2011; Delourme et al., 2013; Wu et al., 2014), is needed for exploitation of heterosis in this crop from a long-term perspective. In this study, we compared the performance of the test hybrids of the inbred lines derived from six *B. napus* × *B. oleracea* interspecific crosses involving four variants of *B. oleracea* and a single *B. napus* line. The design of the production of test hybrids laid out in this research, i.e. the inbred lines were crossed to the *B. napus* parent, allowed us to estimate the effect of the alleles of the different variants of *B. oleracea* for heterosis in *B. napus* canola. We found that seed yield in hybrids in most cases was significantly greater than the *B. napus* parent suggesting that *B. oleracea* alleles contributed to increased seed yield in spring *B. napus* canola hybrids. Of the six population studied, the inbred population derived from the cross involving broccoli gave higher yield than the inbred populations derived from the other five crosses. While evaluating only the inbred lines in larger plot (5.0 m × 1.7–1.8 m) trials, we also found similar results (Nikzad et al., 2019). However, in this study, we found that the test hybrids of the inbred population of broccoli yielded less than the other test hybrid populations; this inbred population, in fact, had the least genetic distance from the *B. napus* parent (data not shown). This indicate that this variant of *B. oleracea* might carry less heterotic alleles for seed yield, or depletion of favorable heterotic alleles might have occurred during the development of this population.

In the present study, we used 95 SSR markers from nine C-genome linkage groups (average 10.7 SSR markers per linkage group). This is not a large number of markers when compared with SNPs; however, limited number (e.g. 18 to 55) of SSRs can provide good information of genetic diversity, as has been reported by several researchers (Wang et al., 2009; Chen et al., 2011; Chen et al., 2017). Genetic distance of the inbred lines, estimated based on the above-mentioned 95 SSR markers, showed a weak or negative (*r* = −0.14) correlation with seed yield in the inbred population; however, this correlation was positive in the test hybrid population as well as with MPH. This indicates that several alleles of *B. oleracea* in homozygous condition gave poor yield in the inbred population; however, at least, some of the alleles were capable of contributing to heterosis through non-additive genetic effect. Involvement of non-additive gene effect for high seed yield in hybrids was also evident from a weak correlation between the performance of the inbred lines and the hybrids, as well as from a weak correlation of the performance of the inbred lines with MPH and NPH. Involvement of both additive and non-additive genes in the genetic control of seed yield in *B. napus* hybrids has also been reported by several researchers (Radoev et al., 2008; Qian et al., 2009). While working with a single *B. oleracea* accession, Rahman et al. (2016) also found the evidence that the alleles of *B. oleracea* contributing to heterosis may not necessarily contribute to seed yield in the inbred lines. The effect of *B. oleracea* alleles on lateness of flowering and maturity and longer duration of flowering is also evident from the positive correlation of genetic distance with these traits in the inbred population. However, several lines flowered and matured earlier than the *B. napus* parent (**Figure 1**) suggesting that, at least, some of the *B. oleracea* alleles can exhibit favorable effect on these traits; identification of these alleles by using high-density markers and molecular mapping approach will be needed for use in a molecular breeding program.

Interspecific hybridization in *Brassica* can induce a number of genetic change in the genome through homoeologous recombination between the chromosomes (Udall et al., 2005; Leflon et al., 2006; Zou et al., 2011) and this can create new genetic variation and exert significant effect on seed yield (Zou et al., 2011; Fu et al., 2012). While working with *B. napus* × *B. rapa* interspecific cross, Fu et al. (2012) found that the novel alleles generated in the progeny of this interspecific cross can contribute to heterosis for seed yield in *B. napus* through allelic and non-allelic interactions. Zou et al. (2010) found improved agronomic performance and strong heterosis for seed yield in hybrids of natural *B. napus* and *B. napus* lines carrying A and C genome contents introgressed from *B. rapa* and *B. carinata*, respectively. Intersubgenomic heterosis in *Brassica* for seed yield in *B. napus* has also been reported by Qian et al. (2005) and Wei et al. (2016). In the present study, the average MPH for seed yield in the six test hybrid populations was 8.5% and about 67% of test hybrids yielded higher than the common *B. napus* parent. Of the six populations we used in this study, greater proportion of the test hybrids of the inbred lines derived from the crosses involving cabbage cv. Balbro and broccoli cv. Premium Crop gave lower yield than the common *B. napus* parent. Multivariate analysis showed that the best hybrid gave about 30% MPH for seed yield and this hybrid originated from the inbred line 74 (**Supplementary Table 1**) derived from the cross involving var. *botrytis* cv. BARI Cauliflower. However, high MPH in individual hybrid was also observed in the populations derived from crosses with Chinese kale and cabbage cv. Badger Shipper. Thus, the wide variation for heterosis observed between the six test hybrid populations might have resulted from the effect of variable alleles from these *B. oleracea* variants. It is also probable that novel genetic variation arose in the progeny of these interspecific crosses might have also contributed to the observed heterosis; further study will be needed to confirm this.

In the present study, backcrossing of the F_1_ to the *B. napus* parent, theoretically, would have diluted the exotic genome content in the BC_1_-derived inbred population, while the F_2_-derived inbred population was expected to have a greater proportion of the genome content of *B. oleracea* and consequently would have resulted greater genetic variation and stronger heterosis in the test hybrids. However, in practice, no significant difference for seed yield was found between the test hybrid populations developed following these two breeding methods. It was also expected that, the BC_1_-derived inbred population will be closer to the common *B. napus* parent than the F_2_ derived population in regards to SSR allele diversity; however, these two populations were genetically quite similar (distance from the *B. napus* parent was 0.47 and 0.49 for the F_2_- and BC_1_-derived populations, respectively). Stronger selection on the F_2_-derived population as compared to the BC_1_-derived population for spring growth habit and the two canola quality traits (zero erucic acid and low glucosinolate) might be one of the reasons for this genetic similarity as well as similar seed yield of the populations developed following these two breeding methods. In contrast, Schelfhout et al. (2008) identified greater number of lines with high seed yield in BC_1_-derived population as compared to F_2_-derived population of *B. napus* × *B. juncea* interspecific cross.

Almost no heterosis was found for seed oil and protein contents in the test hybrid populations of the inbred lines derived from the six interspecific crosses. These two traits are mainly controlled by additive genes (Shen et al., 2005; Variath et al., 2009; for review, see Rahman et al., 2013; Cheng et al., 2016; Chao et al., 2017) which could be the reason for the lack of significant heterosis for these two seed traits as has been found in other studies as well (Grant and Beversdorf, 1985; Rahman et al., 2016). The occurrence of strong positive correlation between the performance of the inbred lines and test hybrids for seed oil and protein contents and weak correlation of these traits with MPH suggests that these two traits are largely controlled by additive genes. Therefore, improvement of the hybrid parent lines will be needed to achieve high oil and protein contents in the hybrid cultivars. However, positive heterosis for seed oil content has also been reported by Shen et al. (2005).

Several inbred lines of the *B. napus* × *B. oleracea* crosses flowered significantly earlier than the common *B. napus* parent indicating that the alleles of the C genome of *B. oleracea* can contribute to earliness in *B. napus*. Test hybrid populations also flowered and matured slightly earlier than their inbred populations. This agree with the results reported by Long et al. (2007) and Rahman et al. (2016); this apparently resulted from partial to complete dominance of some of the genes governing these two quantitative traits (Sernyk and Stefansson, 1983; Cuthbert et al., 2009). Days to flowering in the inbred and test hybrid populations used in the present study didn't show significant correlation with seed yield. This trait has been reported to exhibit a significant negative (Udall et al., 2004; Raman et al., 2016) or a non-significant correlation (Butruille et al., 1999) in spring *B. napus* depending on the types of materials used and test environmental condition. In contrast, the duration of flowering and grain-filling period, respectively, exhibited a significant negative and positive correlation with seed yield. The negative correlation between the duration of flowering and seed yield might have resulted from the failure of the late flowering lines and hybrids to reach physiological maturity at the time of harvest as all plots were desiccated at the same time, and this might had penalized the late flowering ones for seed yield. On the other hand, the longer grain-filling period might had resulted fully developed seeds and, thus, contributed to the positive correlation of this trait with seed yield. Gan et al. (2016) also reported a negative correlation between the duration of flowering and seed yield while a positive correlation between the duration of grain–filling period and seed yield under Canadian environment.

## Conclusion

In conclusion, results from this study showed that the *B. oleracea* alleles introgressed into spring *B. napus* canola inbred lines can exhibit heterosis for seed yield in *B. napus* hybrids. Among the different variants of *B. oleracea* used in this study, cauliflower, Chinese kale and some of the cabbages showed great potential to increase seed yield in spring canola hybrids. However, improvement of the seed yield of the hybrid parent lines will also be needed to increase seed yield in the hybrids as evident from positive correlation of the performance of inbred lines with hybrid yield as well as with NPH. In this regard, alleles introgressed from broccoli can also contribute to hybrid breeding. Thus, introgression of genome content of *B. oleracea* can broaden the genetic base of the C genome of *B. napus* for the development of improved spring *B. napus* canola hybrid cultivars.

## Data Availability Statement

All datasets generated for this study are included in the article/**Supplementary Material**.

## Author Contributions

AN carried out the experiment, collected the data, performed analysis, designed the figures and wrote the first draft of the manuscript which was edited by HR. JP supervised the multivariate analysis. BK, JB, and XW contributed to data collection. BK, JP, R-CY, and HR provided critical feedback and helped to shape the research, data analysis, and read the manuscript. HR conceived the original idea and supervised the research.

## Funding

Financial support for research has been provided by Natural Sciences and Engineering Research Council of Canada (NSERC) and Nutrien Ag Solutions.

## Conflict of Interest

The authors declare that the research was conducted in the absence of any commercial or financial relationships that could be construed as a potential conflict of interest.

## Abbreviation

BLUP, Best linear unbiased prediction; ERS, Edmonton Research Station; LSmeans, least squares means; MPH, mid-parent heterosis; NPH, heterosis over B. napus parent; NIRS, near-infrared reflectance spectroscopy; PCA, principal component analysis; SSR, simple sequence repeat.
