# Supplementary material for: Potential of the C Genome of the Different Variants of Brassica oleracea for Heterosis in Spring B. napus Canola
Source: Front Plant Sci. 2020 Jan 17;10:1691. doi: 10.3389/fpls.2019.01691 (PMC6978715; doi:10.3389/fpls.2019.01691)
Supplement: Supplementary file 1 [file DataSheet_1.docx]

Supplementary Material

# Supplementary Tables

**Supplementary Table 1.** Codes of the F_2_- and BC_1_-derived inbred lines of six *Brassica napus* × *B. oleracea* interspecific crosses

| Inbred line number | Inbred line code | Cross | Breeding method |
| --- | --- | --- | --- |
| 1300-353 | 3 | A04-73NA × *B. oleracea* var. *alboglabra* line NRC-PBI | F |
| 1300-355 | 4 | A04-73NA × *B. oleracea* var. *alboglabra* line NRC-PBI | F |
| 1300-360 | 5 | A04-73NA × *B. oleracea* var. *alboglabra* line NRC-PBI | F |
| 1300-363 | 6 | A04-73NA × *B. oleracea* var. *alboglabra* line NRC-PBI | F |
| 1300-368 | 7 | A04-73NA × *B. oleracea* var. *alboglabra* line NRC-PBI | F |
| 1300-375 | 9 | A04-73NA × *B. oleracea* var. *alboglabra* line NRC-PBI | F |
| 1300-398 | 13 | A04-73NA × *B. oleracea* var. *alboglabra* line NRC-PBI | F |
| 1300-401 | 14 | A04-73NA × *B. oleracea* var. *alboglabra* line NRC-PBI | F |
| 1300-404 | 15 | A04-73NA × *B. oleracea* var. *alboglabra* line NRC-PBI | F |
| 1300-410 | 16 | A04-73NA × *B. oleracea* var. *alboglabra* line NRC-PBI | F |
| 1300-412 | 17 | A04-73NA × *B. oleracea* var. *alboglabra* line NRC-PBI | F |
| 1300-413 | 18 | A04-73NA × *B. oleracea* var. *alboglabra* line NRC-PBI | F |
| 1300-416 | 19 | A04-73NA × *B. oleracea* var. *alboglabra* line NRC-PBI | F |
| 1300-419 | 20 | A04-73NA × *B. oleracea* var. *alboglabra* line NRC-PBI | F |
| 1300-420 | 21 | A04-73NA × *B. oleracea* var. *alboglabra* line NRC-PBI | F |
| 1343-320 | 22 | A04-73NA × *B. oleracea* var. *botrytis* cv. BARI cauliflower | F |
| 1343-321 | 23 | A04-73NA × *B. oleracea* var. *botrytis* cv. BARI cauliflower | F |
| 1343-323 | 24 | A04-73NA × *B. oleracea* var. *botrytis* cv. BARI cauliflower | F |
| 1343-327 | 25 | A04-73NA × *B. oleracea* var. *botrytis* cv. BARI cauliflower | F |
| 1343-329 | 26 | A04-73NA × *B. oleracea* var. *botrytis* cv. BARI cauliflower | F |
| 1343-330 | 27 | A04-73NA × *B. oleracea* var. *botrytis* cv. BARI cauliflower | F |
| 1343-333 | 28 | A04-73NA × *B. oleracea* var. *botrytis* cv. BARI cauliflower | F |
| 1343-336 | 29 | A04-73NA × *B. oleracea* var. *botrytis* cv. BARI cauliflower | F |
| 1343-339 | 30 | A04-73NA × *B. oleracea* var. *botrytis* cv. BARI cauliflower | F |
| 1343-343 | 31 | A04-73NA × *B. oleracea* var. *botrytis* cv. BARI cauliflower | F |
| 1343-349 | 33 | A04-73NA × *B. oleracea* var. *botrytis* cv. BARI cauliflower | F |
| 1343-353 | 35 | A04-73NA × *B. oleracea* var. *botrytis* cv. BARI cauliflower | F |
| 1343-357 | 36 | A04-73NA × *B. oleracea* var. *botrytis* cv. BARI cauliflower | F |
| 1343-360 | 37 | A04-73NA × *B. oleracea* var. *botrytis* cv. BARI cauliflower | F |
| 1343-362 | 38 | A04-73NA × *B. oleracea* var. *botrytis* cv. BARI cauliflower | F |
| 1343-367 | 39 | A04-73NA × *B. oleracea* var. *botrytis* cv. BARI cauliflower | F |
| 1343-368 | 40 | A04-73NA × *B. oleracea* var. *botrytis* cv. BARI cauliflower | F |
| 1676-361 | 41 | A04-73NA × *B. oleracea* var. *alboglabra* line NRC-PBI | BC |
| 1676-363 | 42 | A04-73NA × *B. oleracea* var. *alboglabra* line NRC-PBI | BC |
| 1676-365 | 43 | A04-73NA × *B. oleracea* var. *alboglabra* line NRC-PBI | BC |
| 1676-377 | 44 | A04-73NA × *B. oleracea* var. *alboglabra* line NRC-PBI | BC |
| 1676-380 | 45 | A04-73NA × *B. oleracea* var. *alboglabra* line NRC-PBI | BC |
| 1676-389 | 46 | A04-73NA × *B. oleracea* var. *alboglabra* line NRC-PBI | BC |
| 1676-393 | 47 | A04-73NA × *B. oleracea* var. *alboglabra* line NRC-PBI | BC |
| 1676-402 | 48 | A04-73NA × *B. oleracea* var. *alboglabra* line NRC-PBI | BC |
| 1676-405 | 49 | A04-73NA × *B. oleracea* var. *alboglabra* line NRC-PBI | BC |
| 1676-407 | 50 | A04-73NA × *B. oleracea* var. *alboglabra* line NRC-PBI | BC |
| 1676-409 | 51 | A04-73NA × *B. oleracea* var. *alboglabra* line NRC-PBI | BC |
| 1676-412 | 52 | A04-73NA × *B. oleracea* var. *alboglabra* line NRC-PBI | BC |
| 1676-413 | 53 | A04-73NA × *B. oleracea* var. *alboglabra* line NRC-PBI | BC |
| 1676-416 | 54 | A04-73NA × *B. oleracea* var. *alboglabra* line NRC-PBI | BC |
| 1676-422 | 56 | A04-73NA × *B. oleracea* var. *alboglabra* line NRC-PBI | BC |
| 1676-423 | 57 | A04-73NA × *B. oleracea* var. *alboglabra* line NRC-PBI | BC |
| 1676-427 | 58 | A04-73NA × *B. oleracea* var. *alboglabra* line NRC-PBI | BC |
| 1676-429 | 59 | A04-73NA × *B. oleracea* var. *alboglabra* line NRC-PBI | BC |
| 1676-438 | 61 | A04-73NA × *B. oleracea* var. *alboglabra* line NRC-PBI | BC |
| 1676-442 | 63 | A04-73NA × *B. oleracea* var. *alboglabra* line NRC-PBI | BC |
| 1676-446 | 64 | A04-73NA × *B. oleracea* var. *alboglabra* line NRC-PBI | BC |
| 1677-326 | 65 | A04-73NA × *B. oleracea* var. *botrytis* cv. BARI cauliflower | BC |
| 1677-328 | 66 | A04-73NA × *B. oleracea* var. *botrytis* cv. BARI cauliflower | BC |
| 1677-330 | 67 | A04-73NA × *B. oleracea* var. *botrytis* cv. BARI cauliflower | BC |
| 1677-342 | 70 | A04-73NA × *B. oleracea* var. *botrytis* cv. BARI cauliflower | BC |
| 1677-344 | 71 | A04-73NA × *B. oleracea* var. *botrytis* cv. BARI cauliflower | BC |
| Inbred line number | Inbred line code | Cross | Breeding method |
| 1677-351 | 73 | A04-73NA × *B. oleracea* var. *botrytis* cv. BARI cauliflower | BC |
| 1677-352 | 74 | A04-73NA × *B. oleracea* var. *botrytis* cv. BARI cauliflower | BC |
| 1677-355 | 75 | A04-73NA × *B. oleracea* var. *botrytis* cv. BARI cauliflower | BC |
| 1677-360 | 77 | A04-73NA × *B. oleracea* var. *botrytis* cv. BARI cauliflower | BC |
| 1677-363 | 78 | A04-73NA × *B. oleracea* var. *botrytis* cv. BARI cauliflower | BC |
| 1677-375 | 79 | A04-73NA × *B. oleracea* var. *botrytis* cv. BARI cauliflower | BC |
| 1677-376 | 80 | A04-73NA × *B. oleracea* var. *botrytis* cv. BARI cauliflower | BC |
| 1677-379 | 81 | A04-73NA × *B. oleracea* var. *botrytis* cv. BARI cauliflower | BC |
| 1677-383 | 82 | A04-73NA × *B. oleracea* var. *botrytis* cv. BARI cauliflower | BC |
| 1677-386 | 83 | A04-73NA × *B. oleracea* var. *botrytis* cv. BARI cauliflower | BC |
| 1677-387 | 84 | A04-73NA × *B. oleracea* var. *botrytis* cv. BARI cauliflower | BC |
| 1677-390 | 85 | A04-73NA × *B. oleracea* var. *botrytis* cv. BARI cauliflower | BC |
| 1677-394 | 86 | A04-73NA × *B. oleracea* var. *botrytis* cv. BARI cauliflower | BC |
| 1677-395 | 87 | A04-73NA × *B. oleracea* var. *botrytis* cv. BARI cauliflower | BC |
| 1677-405 | 89 | A04-73NA × *B. oleracea* var. *botrytis* cv. BARI cauliflower | BC |
| 1677-411 | 91 | A04-73NA × *B. oleracea* var. *botrytis* cv. BARI cauliflower | BC |
| 1677-414 | 92 | A04-73NA × *B. oleracea* var. *botrytis* cv. BARI cauliflower | BC |
| 1677-418 | 93 | A04-73NA × *B. oleracea* var. *botrytis* cv. BARI cauliflower | BC |
| 1358-594 | 95 | A04-73NA × *B. oleracea* var. *italica* cv. Premium Crop | F |
| 1358-609 | 96 | A04-73NA × *B. oleracea* var. *italica* cv. Premium Crop | F |
| 1358-615 | 97 | A04-73NA × *B. oleracea* var. *italica* cv. Premium Crop | F |
| 1358-616 | 98 | A04-73NA × *B. oleracea* var. *italica* cv. Premium Crop | F |
| 1358-620 | 99 | A04-73NA × *B. oleracea* var. *italica* cv. Premium Crop | F |
| 1358-623 | 100 | A04-73NA × *B. oleracea* var. *italica* cv. Premium Crop | F |
| 1358-624 | 101 | A04-73NA × *B. oleracea* var. *italica* cv. Premium Crop | F |
| 1358-634 | 102 | A04-73NA × *B. oleracea* var. *italica* cv. Premium Crop | F |
| 1358-635 | 103 | A04-73NA × *B. oleracea* var. *italica* cv. Premium Crop | F |
| 1358-640 | 104 | A04-73NA × *B. oleracea* var. *italica* cv. Premium Crop | F |
| 1358-652 | 105 | A04-73NA × *B. oleracea* var. *italica* cv. Premium Crop | F |
| 1358-656 | 106 | A04-73NA × *B. oleracea* var. *italica* cv. Premium Crop | F |
| 1358-667 | 108 | A04-73NA × *B. oleracea* var. *italica* cv. Premium Crop | F |
| 1358-679 | 109 | A04-73NA × *B. oleracea* var. *italica* cv. Premium Crop | F |
| 1358-685 | 110 | A04-73NA × *B. oleracea* var. *italica* cv. Premium Crop | F |
| 1358-688 | 111 | A04-73NA × *B. oleracea* var. *italica* cv. Premium Crop | F |
| 1358-701 | 113 | A04-73NA × *B. oleracea* var. *italica* cv. Premium Crop | F |
| 1358-703 | 114 | A04-73NA × *B. oleracea* var. *italica* cv. Premium Crop | F |
| 1358-705 | 115 | A04-73NA × *B. oleracea* var. *italica* cv. Premium Crop | F |
| 1358-719 | 118 | A04-73NA × *B. oleracea* var. *italica* cv. Premium Crop | F |
| 1358-720 | 119 | A04-73NA × *B. oleracea* var. *italica* cv. Premium Crop | F |
| 1358-727 | 120 | A04-73NA × *B. oleracea* var. *italica* cv. Premium Crop | F |
| 1358-731 | 121 | A04-73NA × *B. oleracea* var. *italica* cv. Premium Crop | F |
| 1358-739 | 122 | A04-73NA × *B. oleracea* var. *italica* cv. Premium Crop | F |
| 1358-747 | 123 | A04-73NA × *B. oleracea* var. *italica* cv. Premium Crop | F |
| 1358-752 | 124 | A04-73NA × *B. oleracea* var. *italica* cv. Premium Crop | F |
| 1392-300 | 128 | A04-73NA × *B. oleracea* var. *capitata* cv. Balbro | F |
| 1392-303 | 129 | A04-73NA × *B. oleracea* var. *capitata* cv. Balbro | F |
| 1392-305 | 130 | A04-73NA × *B. oleracea* var. *capitata* cv. Balbro | F |
| 1392-306 | 131 | A04-73NA × *B. oleracea* var. *capitata* cv. Balbro | F |
| 1392-312 | 132 | A04-73NA × *B. oleracea* var. *capitata* cv. Balbro | F |
| 1392-313 | 133 | A04-73NA × *B. oleracea* var. *capitata* cv. Balbro | F |
| 1392-319 | 134 | A04-73NA × *B. oleracea* var. *capitata* cv. Balbro | F |
| 1392-320 | 135 | A04-73NA × *B. oleracea* var. *capitata* cv. Balbro | F |
| 1392-324 | 137 | A04-73NA × *B. oleracea* var. *capitata* cv. Balbro | F |
| 1392-325 | 138 | A04-73NA × *B. oleracea* var. *capitata* cv. Balbro | F |
| 1392-327 | 139 | A04-73NA × *B. oleracea* var. *capitata* cv. Balbro | F |
| 1392-329 | 140 | A04-73NA × *B. oleracea* var. *capitata* cv. Balbro | F |
| 1392-337 | 141 | A04-73NA × *B. oleracea* var. *capitata* cv. Balbro | F |
| 1392-339 | 142 | A04-73NA × *B. oleracea* var. *capitata* cv. Balbro | F |
| 1392-342 | 143 | A04-73NA × *B. oleracea* var. *capitata* cv. Balbro | F |
| 1392-345 | 144 | A04-73NA × *B. oleracea* var. *capitata* cv. Balbro | F |
| 1678-263 | 145 | A04-73NA × *B. oleracea* var. *italica* cv. Premium Crop | BC |
| Inbred line number | Inbred line code | Cross | Breeding method |
| 1678-264 | 146 | A04-73NA × *B. oleracea* var. *italica* cv. Premium Crop | BC |
| 1678-265 | 147 | A04-73NA × *B. oleracea* var. *italica* cv. Premium Crop | BC |
| 1678-277 | 150 | A04-73NA × *B. oleracea* var. *italica* cv. Premium Crop | BC |
| 1678-281 | 151 | A04-73NA × *B. oleracea* var. *italica* cv. Premium Crop | BC |
| 1678-285 | 152 | A04-73NA × *B. oleracea* var. *italica* cv. Premium Crop | BC |
| 1678-291 | 154 | A04-73NA × *B. oleracea* var. *italica* cv. Premium Crop | BC |
| 1678-309 | 155 | A04-73NA × *B. oleracea* var. *italica* cv. Premium Crop | BC |
| 1679-354 | 158 | A04-73NA × *B. oleracea* var. *capitata* cv. Balbro | BC |
| 1679-357 | 159 | A04-73NA × *B. oleracea* var. *capitata* cv. Balbro | BC |
| 1679-369 | 160 | A04-73NA × *B. oleracea* var. *capitata* cv. Balbro | BC |
| 1679-377 | 161 | A04-73NA × *B. oleracea* var. *capitata* cv. Balbro | BC |
| 1679-378 | 162 | A04-73NA × *B. oleracea* var. *capitata* cv. Balbro | BC |
| 1679-382 | 164 | A04-73NA × *B. oleracea* var. *capitata* cv. Balbro | BC |
| 1679-399 | 166 | A04-73NA × *B. oleracea* var. *capitata* cv. Balbro | BC |
| 1679-405 | 167 | A04-73NA × *B. oleracea* var. *capitata* cv. Balbro | BC |
| 1679-420 | 168 | A04-73NA × *B. oleracea* var. *capitata* cv. Balbro | BC |
| 1679-430 | 169 | A04-73NA × *B. oleracea* var. *capitata* cv. Balbro | BC |
| 1679-437 | 170 | A04-73NA × *B. oleracea* var. *capitata* cv. Balbro | BC |
| 1679-440 | 171 | A04-73NA × *B. oleracea* var. *capitata* cv. Balbro | BC |
| 1679-442 | 172 | A04-73NA × *B. oleracea* var. *capitata* cv. Balbro | BC |
| 1679-460 | 173 | A04-73NA × *B. oleracea* var. *capitata* cv. Balbro | BC |
| 1679-465 | 174 | A04-73NA × *B. oleracea* var. *capitata* cv. Balbro | BC |
| 1679-470 | 175 | A04-73NA × *B. oleracea* var. *capitata* cv. Balbro | BC |
| 1679-474 | 177 | A04-73NA × *B. oleracea* var. *capitata* cv. Balbro | BC |
| 1679-483 | 178 | A04-73NA × *B. oleracea* var. *capitata* cv. Balbro | BC |
| 1679-486 | 179 | A04-73NA × *B. oleracea* var. *capitata* cv. Balbro | BC |
| 1679-497 | 180 | A04-73NA × *B. oleracea* var. *capitata* cv. Balbro | BC |
| 1679-502 | 181 | A04-73NA × *B. oleracea* var. *capitata* cv. Balbro | BC |
| 1679-506 | 183 | A04-73NA × *B. oleracea* var. *capitata* cv. Balbro | BC |
| 1679-511 | 184 | A04-73NA × *B. oleracea* var. *capitata* cv. Balbro | BC |
| 1679-535 | 185 | A04-73NA × *B. oleracea* var. *capitata* cv. Balbro | BC |
| 1679-541 | 186 | A04-73NA × *B. oleracea* var. *capitata* cv. Balbro | BC |
| 1679-543 | 187 | A04-73NA × *B. oleracea* var. *capitata* cv. Balbro | BC |
| 1362-149 | 188 | A04-73NA × *B. oleracea* var. *capitata* cv. Badger Shipper | F |
| 1362-152 | 189 | A04-73NA × *B. oleracea* var. *capitata* cv. Badger Shipper | F |
| 1362-156 | 191 | A04-73NA × *B. oleracea* var. *capitata* cv. Badger Shipper | F |
| 1362-161 | 193 | A04-73NA × *B. oleracea* var. *capitata* cv. Badger Shipper | F |
| 1362-162 | 194 | A04-73NA × *B. oleracea* var. *capitata* cv. Badger Shipper | F |
| 1362-164 | 195 | A04-73NA × *B. oleracea* var. *capitata* cv. Badger Shipper | F |
| 1362-165 | 196 | A04-73NA × *B. oleracea* var. *capitata* cv. Badger Shipper | F |
| 1362-166 | 197 | A04-73NA × *B. oleracea* var. *capitata* cv. Badger Shipper | F |
| 1362-167 | 198 | A04-73NA × *B. oleracea* var. *capitata* cv. Badger Shipper | F |
| 1362-169 | 199 | A04-73NA × *B. oleracea* var. *capitata* cv. Badger Shipper | F |
| 1362-170 | 200 | A04-73NA × *B. oleracea* var. *capitata* cv. Badger Shipper | F |
| 1362-171 | 201 | A04-73NA × *B. oleracea* var. *capitata* cv. Badger Shipper | F |
| 1362-173 | 202 | A04-73NA × *B. oleracea* var. *capitata* cv. Badger Shipper | F |
| 1362-174 | 203 | A04-73NA × *B. oleracea* var. *capitata* cv. Badger Shipper | F |
| 1362-175 | 204 | A04-73NA × *B. oleracea* var. *capitata* cv. Badger Shipper | F |
| 1362-176 | 205 | A04-73NA × *B. oleracea* var. *capitata* cv. Badger Shipper | F |
| 1362-177 | 206 | A04-73NA × *B. oleracea* var. *capitata* cv. Badger Shipper | F |
| 1362-179 | 207 | A04-73NA × *B. oleracea* var. *capitata* cv. Badger Shipper | F |
| 1362-180 | 208 | A04-73NA × *B. oleracea* var. *capitata* cv. Badger Shipper | F |
| 1363-164 | 209 | A04-73NA × *B. oleracea* var. *capitata* cv. Bindsachsener | F |
| 1363-165 | 210 | A04-73NA × *B. oleracea* var. *capitata* cv. Bindsachsener | F |
| 1363-168 | 211 | A04-73NA × *B. oleracea* var. *capitata* cv. Bindsachsener | F |
| 1363-170 | 212 | A04-73NA × *B. oleracea* var. *capitata* cv. Bindsachsener | F |
| 1363-171 | 213 | A04-73NA × *B. oleracea* var. *capitata* cv. Bindsachsener | F |
| 1363-173 | 214 | A04-73NA × *B. oleracea* var. *capitata* cv. Bindsachsener | F |
| 1363-177 | 215 | A04-73NA × *B. oleracea* var. *capitata* cv. Bindsachsener | F |
| 1363-180 | 217 | A04-73NA × *B. oleracea* var. *capitata* cv. Bindsachsener | F |
| Inbred line number | Inbred line code | Cross | Breeding method |
| 1363-181 | 218 | A04-73NA × *B. oleracea* var. *capitata* cv. Bindsachsener | F |
| 1363-183 | 220 | A04-73NA × *B. oleracea* var. *capitata* cv. Bindsachsener | F |
| 1363-190 | 223 | A04-73NA × *B. oleracea* var. *capitata* cv. Bindsachsener | F |
| 1363-194 | 224 | A04-73NA × *B. oleracea* var. *capitata* cv. Bindsachsener | F |
| 1363-195 | 225 | A04-73NA × *B. oleracea* var. *capitata* cv. Bindsachsener | F |
| 1363-197 | 226 | A04-73NA × *B. oleracea* var. *capitata* cv. Bindsachsener | F |
| 1363-202 | 227 | A04-73NA × *B. oleracea* var. *capitata* cv. Bindsachsener | F |
| 1363-205 | 228 | A04-73NA × *B. oleracea* var. *capitata* cv. Bindsachsener | F |
| 1363-207 | 230 | A04-73NA × *B. oleracea* var. *capitata* cv. Bindsachsener | F |
| 1681-084 | 235 | A04-73NA × *B. oleracea* var. *capitata* cv. Badger Shipper | BC |
| 1681-083 | 234 | A04-73NA × *B. oleracea* var. *capitata* cv. Badger Shipper | BC |
| 1681-086 | 237 | A04-73NA × *B. oleracea* var. *capitata* cv. Badger Shipper | BC |
| 1681-090 | 238 | A04-73NA × *B. oleracea* var. *capitata* cv. Badger Shipper | BC |
| 1681-091 | 239 | A04-73NA × *B. oleracea* var. *capitata* cv. Badger Shipper | BC |
| 1681-092 | 240 | A04-73NA × *B. oleracea* var. *capitata* cv. Badger Shipper | BC |
| 1681-096 | 241 | A04-73NA × *B. oleracea* var. *capitata* cv. Badger Shipper | BC |
| 1681-097 | 242 | A04-73NA × *B. oleracea* var. *capitata* cv. Badger Shipper | BC |
| 1681-100 | 244 | A04-73NA × *B. oleracea* var. *capitata* cv. Badger Shipper | BC |
| 1681-101 | 245 | A04-73NA × *B. oleracea* var. *capitata* cv. Badger Shipper | BC |
| 1681-102 | 246 | A04-73NA × *B. oleracea* var. *capitata* cv. Badger Shipper | BC |
| 1681-103 | 247 | A04-73NA × *B. oleracea* var. *capitata* cv. Badger Shipper | BC |
| 1681-104 | 248 | A04-73NA × *B. oleracea* var. *capitata* cv. Badger Shipper | BC |
| 1681-105 | 249 | A04-73NA × *B. oleracea* var. *capitata* cv. Badger Shipper | BC |
| 1682-099 | 250 | A04-73NA × *B. oleracea* var. *capitata* cv. Bindsachsener | BC |
| 1682-100 | 251 | A04-73NA × *B. oleracea* var. *capitata* cv. Bindsachsener | BC |
| 1682-101 | 252 | A04-73NA × *B. oleracea* var. *capitata* cv. Bindsachsener | BC |
| 1682-102 | 253 | A04-73NA × *B. oleracea* var. *capitata* cv. Bindsachsener | BC |
| 1682-103 | 254 | A04-73NA × *B. oleracea* var. *capitata* cv. Bindsachsener | BC |
| 1682-104 | 255 | A04-73NA × *B. oleracea* var. *capitata* cv. Bindsachsener | BC |
| 1682-105 | 256 | A04-73NA × *B. oleracea* var. *capitata* cv. Bindsachsener | BC |
| 1682-108 | 257 | A04-73NA × *B. oleracea* var. *capitata* cv. Bindsachsener | BC |
| 1682-113 | 258 | A04-73NA × *B. oleracea* var. *capitata* cv. Bindsachsener | BC |
| 1682-120 | 259 | A04-73NA × *B. oleracea* var. *capitata* cv. Bindsachsener | BC |
| 1682-128 | 262 | A04-73NA × *B. oleracea* var. *capitata* cv. Bindsachsener | BC |
| 1682-130 | 263 | A04-73NA × *B. oleracea* var. *capitata* cv. Bindsachsener | BC |
| 1682-131 | 264 | A04-73NA × *B. oleracea* var. *capitata* cv. Bindsachsener | BC |
| 1682-133 | 265 | A04-73NA × *B. oleracea* var. *capitata* cv. Bindsachsener | BC |
| 1682-138 | 267 | A04-73NA × *B. oleracea* var. *capitata* cv. Bindsachsener | BC |
| 1682-140 | 268 | A04-73NA × *B. oleracea* var. *capitata* cv. Bindsachsener | BC |
| 1682-143 | 269 | A04-73NA × *B. oleracea* var. *capitata* cv. Bindsachsener | BC |
| 1682-145 | 270 | A04-73NA × *B. oleracea* var. *capitata* cv. Bindsachsener | BC |
| 1682-147 | 271 | A04-73NA × *B. oleracea* var. *capitata* cv. Bindsachsener | BC |
| 1682-149 | 272 | A04-73NA × *B. oleracea* var. *capitata* cv. Bindsachsener | BC |
| 1682-150 | 273 | A04-73NA × *B. oleracea* var. *capitata* cv. Bindsachsener | BC |
| 1682-152 | 274 | A04-73NA × *B. oleracea* var. *capitata* cv. Bindsachsener | BC |
| 1682-154 | 275 | A04-73NA × *B. oleracea* var. *capitata* cv. Bindsachsener | BC |
| 1682-155 | 276 | A04-73NA × *B. oleracea* var. *capitata* cv. Bindsachsener | BC |
| 1682-156 | 277 | A04-73NA × *B. oleracea* var. *capitata* cv. Bindsachsener | BC |
| 1682-158 | 278 | A04-73NA × *B. oleracea* var. *capitata* cv. Bindsachsener | BC |

**Supplementary Table 2.** Least square means ± SE of the inbred lines derived from six *Brassica napus* × *B. oleracea* interspecific crosses, their test-hybrids, mid-parent heterosis (MPH), and heterosis over the common *B. napus* parent (NPH) for different agronomic and seed quality traits.

| Cross^1^ | Pop. type | Days to flowering | Duration of flowering (day) | Days to maturity | Grain-filling period (day) | Plant height (cm) | Seed yield (kg ha^-1^) | Seed oil (%) | Seed protein (%) |
| --- | --- | --- | --- | --- | --- | --- | --- | --- | --- |
| Ol.alb.nrc | Inbred | 48.6 ± 0.6 | 28.2 ± 2.8 | 106.3 ± 1.3 | 29.1 ± 5.4 | 114.1 ± 7.7 | 3387.2 ± 495.6 | 48.0 ± 1.3 | 24.0 ± 1.2 |
|  | Hybrid | 47.6 ± 0.6 | 27.3 ± 2.8 | 106.0 ± 1.3 | 29.7 ± 5.4 | 119.1 ± 7.7 | 3932.1 ± 495.6 | 48.6 ± 1.3 | 23.9 ± 1.2 |
|  | *t test^2^* | *** | NS | NS | NS | *** | *** | * | NS |
|  | MPH | -1.6 ± 0.9 | 1.5 ± 3.8 | 0.1 ± 0.4 | 0.3 ± 2.6 | 1.0 ± 1.1 | 11.1 ± 2.2 | -0.1 ± 0.3 | 0.1 ± 0.6 |
|  | NPH | -1.3 ± 0.9 | 6.3 ± 3.8 | 0.4 ± 0.4 | -2.2 ± 2.6 | -2.1 ± 1.1 | 4.8 ± 2.2 | -1.5 ± 0.3 | 0.9 ± 0.6 |
|  | *t test^3^* | NS | *** | NS | NS | *** | *** | *** | NS |
| Ol.bot.cau | Inbred | 48.3 ± 0.6 | 28.1 ± 2.8 | 106.2 ± 1.3 | 29.1 ± 5.4 | 114.4 ± 7.7 | 3402.0 ± 495.6 | 48.4 ± 1.3 | 24.1 ± 1.2 |
|  | Hybrid | 47.3 ± 0.6 | 27.2 ± 2.8 | 105.8 ± 1.3 | 29.8 ± 5.4 | 118.3 ± 7.7 | 3936.2 ± 495.6 | 48.8 ± 1.3 | 23.9 ± 1.2 |
|  | *t test* | *** | NS | NS | NS | *** | *** | NS | NS |
|  | MPH | -2.0 ± 0.9 | 1.4 ± 3.8 | -0.1 ± 0.4 | 0.4 ± 2.6 | 0.2 ± 1.1 | 10.7 ± 2.1 | -0.2 ± 0.3 | 0.2 ± 0.6 |
|  | NPH | -1.9 ± 0.9 | 6.0 ± 3.8 | 0.2 ± 0.4 | -2.0 ± 2.6 | -2.8 ± 1.1 | 4.3 ± 2.1 | -1.1 ± 0.3 | 1.1 ± 0.6 |
|  | *t test* | NS | *** | NS | NS | *** | *** | *** | NS |
| Ol.cap.bad | Inbred | 48.6 ± 0.6 | 30.4 ± 2.8 | 106.3 ± 1.3 | 27.4 ± 5.4 | 118.9 ± 7.7 | 3260.2 ± 495.7 | 47.0 ± 1.3 | 25.3 ± 1.2 |
|  | Hybrid | 47.3 ± 0.6 | 28.0 ± 2.8 | 105.5 ± 1.3 | 29.1 ± 5.4 | 122.8 ± 7.7 | 3834.7 ± 495.7 | 47.7 ± 1.3 | 24.8 ± 1.2 |
|  | *t test* | *** | *** | *** | *** | *** | *** | *** | *** |
|  | MPH | -1.8 ± 0.9 | -1.5 ± 3.8 | -0.3 ± 0.5 | 1.3 ± 2.6 | 0.7 ± 1.1 | 10.3 ± 2.2 | -0.2 ± 0.3 | 0.0 ± 0.7 |
|  | NPH | -0.9 ± 0.9 | 5.6 ± 3.8 | 0.3 ± 0.5 | -4.1 ± 2.6 | -1.6 ± 1.1 | 2.9 ± 2.2 | -1.9 ± 0.3 | 2.4 ± 0.7 |
|  | *t test* | NS | *** | *** | *** | *** | *** | *** | *** |
| Ol.cap.bal | Inbred | 48.8 ± 0.6 | 28.1 ± 2.8 | 106.5 ± 1.3 | 28.1 ± 5.4 | 115.4 ± 7.7 | 3339.0 ± 495.5 | 47.3 ± 1.3 | 24.7 ± 1.2 |
|  | Hybrid | 47.7 ± 0.6 | 27.8 ± 2.8 | 106.1 ± 1.3 | 29.0 ± 5.4 | 117.8 ± 7.7 | 3693.8 ± 495.6 | 47.6 ± 1.3 | 24.6 ± 1.2 |
|  | *t test* | *** | NS | NS | ** | * | *** | NS | NS |
|  | MPH | -1.2 ± 0.9 | 2.2 ± 3.8 | 0.1 ± 0.4 | -0.8 ± 2.6 | 0.5 ± 1.1 | 6.3 ± 2.1 | -0.4 ± 0.3 | 0.5 ± 0.6 |
|  | NPH | -0.1 ± 0.9 | 6.1 ± 3.8 | 0.5 ± 0.4 | -4.5 ± 2.6 | -1.0 ± 1.1 | 1.5 ± 2.1 | -1.5 ± 0.3 | 1.3 ± 0.6 |
|  | *t test* | ** | *** | ** | *** | ** | ** | *** | NS |
| Ol.cap.bin | Inbred | 47.1 ± 0.6 | 29.3 ± 2.8 | 105.6 ± 1.3 | 28.5 ± 5.4 | 118.7 ± 7.7 | 3333.2 ± 495.5 | 47.8 ± 1.3 | 25.2 ± 1.2 |
|  | Hybrid | 46.7 ± 0.6 | 27.9 ± 2.8 | 105.3 ± 1.3 | 29.4 ± 5.4 | 122.0 ± 7.7 | 3809.6 ± 495.5 | 48.1 ± 1.3 | 24.9 ± 1.2 |
|  | *t test* | NS | ** | NS | ** | *** | *** | NS | * |
|  | MPH | -1.6 ± 0.9 | 0.4 ± 3.8 | -0.2 ± 0.4 | -0.2 ± 2.6 | 0.0 ± 1.1 | 8.3 ± 2.1 | -0.4 ± 0.3 | 0.7 ± 0.6 |
|  | NPH | -2.1 ± 0.9 | 5.8 ± 3.8 | 0.1 ± 0.4 | -3.3 ± 2.6 | -2.5 ± 1.1 | 1.6 ± 2.1 | -1.3 ± 0.3 | 2.9 ± 0.6 |
|  | *t test* | NS | *** | NS | ** | *** | *** | *** | *** |
| Ol.ita.pre | Inbred | 47.9 ± 0.6 | 27.9 ± 2.8 | 105.8 ± 1.3 | 28.4 ± 5.4 | 115.2 ± 7.7 | 3426.5 ± 495.7 | 47.8 ± 1.3 | 24.6 ± 1.2 |
|  | Hybrid | 47.5 ± 0.6 | 28.2 ± 2.8 | 106.0 ± 1.3 | 28.6 ± 5.4 | 117.9 ± 7.7 | 3668.1 ± 495.7 | 47.8 ± 1.3 | 24.5 ± 1.2 |
|  | *t test* | NS | NS | NS | NS | * | *** | NS | NS |
|  | MPH | -0.6 ± 0.9 | 4.1 ± 3.8 | 0.3 ± 0.5 | -2.9 ± 2.6 | 0.3 ± 1.1 | 4.0 ± 2.2 | -0.6 ± 0.3 | 0.5 ± 0.7 |
|  | NPH | -0.3 ± 0.9 | 7.4 ± 3.8 | 0.4 ± 0.5 | -6.1 ± 2.6 | -1.8 ± 1.1 | 0.2 ± 2.2 | -1.2 ± 0.3 | 1.2 ± 0.7 |
|  | *t test* | NS | * | NS | * | *** | NS | NS | NS |
| A04-73NA | | 47.9 ± 0.6 | 26 ± 2.8 | 105.5 ± 1.3 | 30.2 ± 5.4 | 122.2 ± 7.7 | 3716.6 ± 495.1 | 48.8 ± 1.3 | 24 ± 1.2 |

^1^ Ol.alb.nrc = *B. napus* (A04-73NA) × *B. oleracea* var. *alboglabra* line NRC-PBI; Ol.bot.cau = *B. napus* (A04-73NA) × *B. oleracea* var. *botrytis* cv. BARI cauliflower; Ol.cap.bad = *B. napus* (A04-73NA) × *B. oleracea* var. *capitata* cv. Badger Shipper; Ol.cap.bin = *B. napus* (A04-73NA) × *B.* *oleracea* var*. capitata* cv. Bindsachsener; Ol.cap.bal = *B. napus* (A04-73NA) × *B.* *oleracea* var. *capitata* cv. Balbro; Ol.ita.pre = A04-73NA × *B. oleracea* var. *italica* cv. Premium Crop; ^2^ *t test*: Inbred vs. hybrid; ^3^ *t test*: MPH vs. NPH

**Supplementary Table 3.** Least square means ± SE of the inbred populations derived from two breading methods (F_2_- and BC_1_-derived), their test-hybrids, mid-parent heterosis (MPH), and heterosis over the common *B. napus* parent (NPH) for different agronomic and seed quality traits

| Breeding method^1^ | Pop. type^2^ | Days to flowering | Duration of flowering (day) | Days to maturity | Grain-filling period (day) | Plant height (cm) | Seed yield (kg ha^-1^) | Seed oil (%) | Seed protein (%) |
| --- | --- | --- | --- | --- | --- | --- | --- | --- | --- |
| F | Inbred | 48.5 ± 0.6 | 28.9 ± 2.8 | 106.3 ± 1.3 | 28.2 ± 5.4 | 116.9 ± 7.7 | 3359.1 ± 495.3 | 47.8 ± 1.3 | 24.7 ± 1.2 |
|  | Hybrid | 47.5 ± 0.6 | 27.9 ± 2.8 | 105.9 ± 1.3 | 29.1 ± 5.4 | 119.9 ± 7.7 | 3795.1 ± 495.3 | 48.1 ± 1.3 | 24.5 ± 1.2 |
|  | *t test^3^* | *** | *** | ** | *** | *** | *** | ** | * |
|  | MPH | -1.3 ± 0.8 | 0.0 ± 0.4 | 0.0 ± 0.4 | -0.7 ± 2.6 | 0.3 ± 1.1 | 8.0 ± 2.1 | -0.3 ± 0.3 | 0.3 ± 0.6 |
|  | NPH | -0.6 ± 0.8 | 6.8 ± 3.7 | 0.4 ± 0.4 | -4.4 ± 2.6 | -1.7 ± 1.1 | 2.5 ± 2.1 | -1.3 ± 0.3 | 1.6 ± 0.6 |
|  | *t test^4^* | ** | *** | *** | *** | *** | *** | *** | *** |
| BC | Inbred | 47.9 ± 0.6 | 28.4 ± 2.8 | 106.0 ± 1.3 | 28.7 ± 5.4 | 115.5 ± 7.7 | 3357.6 ± 495.2 | 47.7 ± 1.3 | 24.6 ± 1.2 |
|  | Hybrid | 47.1 ± 0.6 | 27.5 ± 2.8 | 105.7 ± 1.3 | 29.5 ± 5.4 | 119.3 ± 7.7 | 3828.5 ± 495.2 | 48.1 ± 1.3 | 24.4 ± 1.2 |
|  | *t test* | *** | *** | NS | *** | *** | *** | *** | * |
|  | MPH | -1.7 ± 0.8 | 1.2 ± 3.7 | 0.0 ± 0.4 | 0.1 ± 2.6 | 0.5 ± 1.1 | 8.8 ± 2 | -0.3 ± 0.3 | 0.4 ± 0.6 |
|  | NPH | -1.6 ± 0.8 | 5.6 ± 3.7 | 0.2 ± 0.4 | -2.9 ± 2.6 | -2.2 ± 1.1 | 2.7 ± 2 | -1.5 ± 0.3 | 1.7 ± 0.6 |
|  | *t test* | NS | *** | *** | *** | *** | *** | *** | *** |
| CV (%) | Inbred | 5.2 | 16.1 | 2.6 | 22.3 | 12.2 | 28.1 | 5.4 | 9.7 |
|  | Hybrid | 4.0 | 12.2 | 2.4 | 19.9 | 12.2 | 24.7 | 4.6 | 9.1 |

^*^ Significant at P < 0.05, ^**^ Significant at P < 0.01, ^***^ Significant at P < 0.001, ^NS^ Not significant

^1^ F = F_2_-derived populations of the cross; BC = BC_1_ (F_1_ × *B. napus*)-derived population of the cross

^2^ Pop. type = population type: Inbred = inbred line population; Hybrid = test hybrid population; MPV = mid-parent value; MPH = mid-parent heterosis; NPH = heterosis over common parent *B. napus* canola

^3^ *t test*: Inbred vs. hybrid

^4^ *t test*: MPH vs. NPH

**Supplementary Table 4.** Comparisons of the least square mean (± SE) values of the inbred lines, derived from six *Brassica napus* × *B. oleracea* interspecific crosses, their common *B. napus* parent (A04-73NA), test-hybrids, mid-parent heterosis (MPH), and heterosis over the common *B. napus* parent (NPH) for different agronomic and seed quality traits

| Comparisons | | Days to flowering | Duration of flowering (day) | Days to maturity | Grain-filling period (day) | Plant height (cm) | Seed yield (kg ha^-1^) | Seed oil (%) | Seed protein (%) |
| --- | --- | --- | --- | --- | --- | --- | --- | --- | --- |
| A04-73NA | Ol.alb.nrc-Inbred | -0.7 ± 0.2** | -2.2 ± 0.3*** | -0.8 ± 0.1*** | 1.2 ± 0.2*** | 8.1 ± 0.6*** | 329.5 ± 27.0*** | 0.8 ± 0.1*** | 0.0 ± 0.1NS |
| A04-73NA | Ol.alb.nrc-Hybrid | 0.3 ± 0.2NS | -1.3 ± 0.3*** | -0.5 ± 0.1* | 0.5 ± 0.2NS | 3.1 ± 0.6*** | -215.5 ± 27.0*** | 0.2 ± 0.1NS | 0.2 ± 0.1NS |
| A04-73NA | Ol.bot.cau-Inbred | -0.5 ± 0.2NS | -2.1 ± 0.3*** | -0.7 ± 0.1*** | 1.1 ± 0.2*** | 7.8 ± 0.5*** | 314.6 ± 25.8*** | 0.4 ± 0.1* | 0.0 ± 0.1NS |
| A04-73NA | Ol.bot.cau-Hybrid | 0.6 ± 0.2* | -1.2 ± 0.3*** | -0.3 ± 0.1NS | 0.4 ± 0.2NS | 3.9 ± 0.5*** | -219.6 ± 25.8*** | 0.0 ± 0.1NS | 0.1 ± 0.1NS |
| A04-73NA | Ol.cap.bad-Inbred | -0.7 ± 0.2** | -4.4 ± 0.3*** | -0.9 ± 0.1*** | 2.8 ± 0.2*** | 3.4 ± 0.6*** | 456.4 ± 28.1*** | 1.8 ± 0.1*** | -1.3 ± 0.1*** |
| A04-73NA | Ol.cap.bad-Hybrid | 0.6 ± 0.2* | -2.0 ± 0.3*** | 0.0 ± 0.1NS | 1.1 ± 0.2*** | -0.6 ± 0.6NS | -118.0 ± 28.1** | 1.1 ± 0.1*** | -0.7 ± 0.1*** |
| A04-73NA | Ol.cap.bal-Inbred | -1.0 ± 0.2*** | -2.1 ± 0.2*** | -1.0 ± 0.1*** | 2.1 ± 0.2*** | 6.8 ± 0.5*** | 377.6 ± 25.2*** | 1.5 ± 0.1*** | -0.6 ± 0.1*** |
| A04-73NA | Ol.cap.bal-Hybrid | 0.2 ± 0.2NS | -1.7 ± 0.2*** | -0.7 ± 0.1*** | 1.2 ± 0.2*** | 4.5 ± 0.5*** | 22.9 ± 25.3NS | 1.2 ± 0.1*** | -0.5 ± 0.1*** |
| A04-73NA | Ol.cap.bin-Inbred | 0.7 ± 0.2*** | -3.2 ± 0.2*** | -0.2 ± 0.1NS | 1.7 ± 0.2*** | 3.5 ± 0.5*** | 383.4 ± 25.0*** | 1.0 ± 0.1*** | -1.2 ± 0.1*** |
| A04-73NA | Ol.cap.bin-Hybrid | 1.1 ± 0.2*** | -1.8 ± 0.2*** | 0.2 ± 0.1NS | 0.9 ± 0.2*** | 0.3 ± 0.5NS | -92.9 ± 25.1* | 0.7 ± 0.1*** | -0.8 ± 0.1*** |
| A04-73NA | Ol.ita.pre-Inbred | 0.0 ± 0.2NS | -1.9 ± 0.3*** | -0.3 ± 0.1NS | 1.8 ± 0.2*** | 7.0 ± 0.6*** | 290.2 ± 27.6*** | 1.0 ± 0.1*** | -0.5 ± 0.1*** |
| A04-73NA | Ol.ita.pre-Hybrid | 0.4 ± 0.2NS | -2.2 ± 0.3*** | -0.6 ± 0.1** | 1.6 ± 0.2*** | 4.3 ± 0.6*** | 48.5 ± 27.6NS | 1.0 ± 0.1*** | -0.5 ± 0.1*** |
| Ol.alb.nrc-Inbred | Ol.alb.nrc-Hybrid | 1.0 ± 0.2*** | 0.9 ± 0.3NS | 0.3 ± 0.2NS | -0.7 ± 0.2NS | -5.0 ± 0.7*** | -544.9 ± 35.4*** | -0.6 ± 0.1** | 0.2 ± 0.1NS |
| Ol.alb.nrc-Inbred | Ol.bot.cau-Inbred | 0.2 ± 0.2NS | 0.1 ± 0.3NS | 0.1 ± 0.2NS | 0.0 ± 0.2NS | -0.3 ± 0.7NS | -14.9 ± 34.5NS | -0.4 ± 0.1NS | -0.1 ± 0.1NS |
| Ol.alb.nrc-Inbred | Ol.bot.cau-Hybrid | 1.3 ± 0.2*** | 1.0 ± 0.3NS | 0.5 ± 0.2NS | -0.7 ± 0.2NS | -4.2 ± 0.7*** | -549.0 ± 34.5*** | -0.8 ± 0.1*** | 0.1 ± 0.1NS |
| Ol.alb.nrc-Inbred | Ol.cap.bad-Inbred | 0.0 ± 0.2NS | -2.2 ± 0.4*** | 0.0 ± 0.2NS | 1.7 ± 0.2*** | -4.7 ± 0.8*** | 127.0 ± 36.3* | 1.0 ± 0.1*** | -1.3 ± 0.1*** |
| Ol.alb.nrc-Inbred | Ol.cap.bad-Hybrid | 1.3 ± 0.2*** | 0.2 ± 0.4NS | 0.8 ± 0.2** | 0.0 ± 0.2NS | -8.7 ± 0.8*** | -447.5 ± 36.3*** | 0.2 ± 0.1NS | -0.8 ± 0.1*** |
| Ol.alb.nrc-Inbred | Ol.cap.bal-Inbred | -0.3 ± 0.2NS | 0.1 ± 0.3NS | -0.2 ± 0.2NS | 0.9 ± 0.2** | -1.3 ± 0.7NS | 48.2 ± 34.1NS | 0.7 ± 0.1*** | -0.7 ± 0.1*** |
| Ol.alb.nrc-Inbred | Ol.cap.bal-Hybrid | 0.9 ± 0.2*** | 0.5 ± 0.3NS | 0.2 ± 0.2NS | 0.1 ± 0.2NS | -3.6 ± 0.7*** | -306.6 ± 34.2*** | 0.3 ± 0.1NS | -0.6 ± 0.1*** |
| Ol.alb.nrc-Inbred | Ol.cap.bin-Inbred | 1.4 ± 0.2*** | -1.0 ± 0.3NS | 0.7 ± 0.2* | 0.5 ± 0.2NS | -4.6 ± 0.7*** | 54.0 ± 34.0NS | 0.2 ± 0.1NS | -1.2 ± 0.1*** |
| Ol.alb.nrc-Inbred | Ol.cap.bin-Hybrid | 1.8 ± 0.2*** | 0.4 ± 0.3NS | 1.0 ± 0.2*** | -0.3 ± 0.2NS | -7.8 ± 0.7*** | -422.4 ± 34.0*** | -0.1 ± 0.1NS | -0.9 ± 0.1*** |
| Ol.alb.nrc-Inbred | Ol.ita.pre-Inbred | 0.7 ± 0.2NS | 0.3 ± 0.4NS | 0.5 ± 0.2NS | 0.6 ± 0.2NS | -1.1 ± 0.7NS | -39.3 ± 35.9NS | 0.2 ± 0.1NS | -0.6 ± 0.1*** |
| Ol.alb.nrc-Inbred | Ol.ita.pre-Hybrid | 1.1 ± 0.2*** | 0.0 ± 0.4NS | 0.3 ± 0.2NS | 0.5 ± 0.2NS | -3.8 ± 0.7*** | -280.9 ± 35.9*** | 0.2 ± 0.1NS | -0.5 ± 0.1*** |
| Ol.alb.nrc-Hybrid | Ol.bot.cau-Inbred | -0.7 ± 0.2* | -0.8 ± 0.3NS | -0.2 ± 0.2NS | 0.6 ± 0.2NS | 4.7 ± 0.7*** | 530.1 ± 34.5*** | 0.1 ± 0.1NS | -0.2 ± 0.1NS |
| Ol.alb.nrc-Hybrid | Ol.bot.cau-Hybrid | 0.3 ± 0.2NS | 0.1 ± 0.3NS | 0.2 ± 0.2NS | -0.1 ± 0.2NS | 0.8 ± 0.7NS | -4.1 ± 34.5NS | -0.2 ± 0.1NS | 0.0 ± 0.1NS |
| Ol.alb.nrc-Hybrid | Ol.cap.bad-Inbred | -1.0 ± 0.2*** | -3.1 ± 0.4*** | -0.3 ± 0.2NS | 2.4 ± 0.2*** | 0.2 ± 0.8NS | 671.9 ± 36.3*** | 1.6 ± 0.1*** | -1.5 ± 0.1*** |
| Ol.alb.nrc-Hybrid | Ol.cap.bad-Hybrid | 0.3 ± 0.2NS | -0.6 ± 0.4NS | 0.5 ± 0.2NS | 0.6 ± 0.2NS | -3.7 ± 0.8*** | 97.4 ± 36.3NS | 0.8 ± 0.1*** | -0.9 ± 0.1*** |
| Ol.alb.nrc-Hybrid | Ol.cap.bal-Inbred | -1.2 ± 0.2*** | -0.8 ± 0.3NS | -0.5 ± 0.2NS | 1.6 ± 0.2*** | 3.7 ± 0.7*** | 593.1 ± 34.1*** | 1.3 ± 0.1*** | -0.8 ± 0.1*** |
| Ol.alb.nrc-Hybrid | Ol.cap.bal-Hybrid | -0.1 ± 0.2NS | -0.4 ± 0.3NS | -0.1 ± 0.2NS | 0.7 ± 0.2NS | 1.3 ± 0.7NS | 238.3 ± 34.2*** | 0.9 ± 0.1*** | -0.7 ± 0.1*** |
| Ol.alb.nrc-Hybrid | Ol.cap.bin-Inbred | 0.4 ± 0.2NS | -1.9 ± 0.3*** | 0.4 ± 0.2NS | 1.2 ± 0.2*** | 0.4 ± 0.7NS | 598.9 ± 34.0*** | 0.7 ± 0.1*** | -1.4 ± 0.1*** |
| Ol.alb.nrc-Hybrid | Ol.cap.bin-Hybrid | 0.9 ± 0.2** | -0.5 ± 0.3NS | 0.7 ± 0.2** | 0.4 ± 0.2NS | -2.9 ± 0.7** | 122.5 ± 34.0* | 0.5 ± 0.1* | -1.0 ± 0.1*** |
| Ol.alb.nrc-Hybrid | Ol.ita.pre-Inbred | -0.3 ± 0.2NS | -0.6 ± 0.4NS | 0.2 ± 0.2NS | 1.3 ± 0.2*** | 3.9 ± 0.7*** | 505.6 ± 35.9*** | 0.8 ± 0.1*** | -0.7 ± 0.1*** |
| Ol.alb.nrc-Hybrid | Ol.ita.pre-Hybrid | 0.1 ± 0.2NS | -0.8 ± 0.4NS | 0.0 ± 0.2NS | 1.1 ± 0.2*** | 1.2 ± 0.7NS | 264 ± 35.9*** | 0.8 ± 0.1*** | -0.7 ± 0.1*** |
| Ol.bot.cau-Inbred | Ol.bot.cau-Hybrid | 1.0 ± 0.2*** | 0.9 ± 0.3NS | 0.4 ± 0.2NS | -0.7 ± 0.2NS | -3.9 ± 0.7*** | -534.2 ± 33.6*** | -0.3 ± 0.1NS | 0.2 ± 0.1NS |
| Ol.bot.cau-Inbred | Ol.cap.bad-Inbred | -0.2 ± 0.2NS | -2.3 ± 0.3*** | -0.2 ± 0.2NS | 1.7 ± 0.2*** | -4.5 ± 0.7*** | 141.8 ± 35.4** | 1.5 ± 0.1*** | -1.3 ± 0.1*** |
| Ol.bot.cau-Inbred | Ol.cap.bad-Hybrid | 1.0 ± 0.2*** | 0.1 ± 0.3NS | 0.7 ± 0.2* | 0.0 ± 0.2NS | -8.4 ± 0.7*** | -432.7 ± 35.4*** | 0.7 ± 0.1*** | -0.7 ± 0.1*** |
| Ol.bot.cau-Inbred | Ol.cap.bal-Inbred | -0.5 ± 0.2NS | 0.0 ± 0.3NS | -0.3 ± 0.2NS | 1.0 ± 0.2*** | -1.0 ± 0.7NS | 63.0 ± 33.2NS | 1.1 ± 0.1*** | -0.6 ± 0.1*** |
| Ol.bot.cau-Inbred | Ol.cap.bal-Hybrid | 0.7 ± 0.2* | 0.3 ± 0.3NS | 0.1 ± 0.2NS | 0.1 ± 0.2NS | -3.4 ± 0.7*** | -291.7 ± 33.3*** | 0.8 ± 0.1*** | -0.5 ± 0.1*** |
| Ol.bot.cau-Inbred | Ol.cap.bin-Inbred | 1.2 ± 0.2*** | -1.2 ± 0.3* | 0.5 ± 0.2NS | 0.6 ± 0.2NS | -4.3 ± 0.7*** | 68.8 ± 33.0NS | 0.6 ± 0.1*** | -1.1 ± 0.1*** |
| Ol.bot.cau-Inbred | Ol.cap.bin-Hybrid | 1.6 ± 0.2*** | 0.2 ± 0.3NS | 0.9 ± 0.2*** | -0.3 ± 0.2NS | -7.6 ± 0.7*** | -407.5 ± 33.1*** | 0.4 ± 0.1NS | -0.8 ± 0.1*** |
| Ol.bot.cau-Inbred | Ol.ita.pre-Inbred | 0.4 ± 0.2NS | 0.2 ± 0.3NS | 0.4 ± 0.2NS | 0.7 ± 0.2NS | -0.8 ± 0.7NS | -24.4 ± 35.1NS | 0.7 ± 0.1*** | -0.5 ± 0.1*** |
| Ol.bot.cau-Inbred | Ol.ita.pre-Hybrid | 0.9 ± 0.2** | -0.1 ± 0.3NS | 0.2 ± 0.2NS | 0.5 ± 0.2NS | -3.5 ± 0.7*** | -266.1 ± 35.1*** | 0.6 ± 0.1** | -0.5 ± 0.1*** |
| Comparisons | | Days to flowering | Duration of flowering (day) | Days to maturity | Grain-filling period (day) | Plant height (cm) | Seed yield (kg ha^-1^) | Seed oil (%) | Seed protein (%) |
| Ol.bot.cau-Hybrid | Ol.cap.bad-Inbred | -1.3 ± 0.2*** | -3.2 ± 0.3*** | -0.5 ± 0.2NS | 2.4 ± 0.2*** | -0.5 ± 0.7NS | 676.0 ± 35.4*** | 1.8 ± 0.1*** | -1.4 ± 0.1*** |
| Ol.bot.cau-Hybrid | Ol.cap.bad-Hybrid | 0.0 ± 0.2NS | -0.8 ± 0.3NS | 0.3 ± 0.2NS | 0.7 ± 0.2NS | -4.5 ± 0.7*** | 101.5 ± 35.4NS | 1.0 ± 0.1*** | -0.9 ± 0.1*** |
| Ol.bot.cau-Hybrid | Ol.cap.bal-Inbred | -1.5 ± 0.2*** | -0.9 ± 0.3NS | -0.7 ± 0.2* | 1.7 ± 0.2*** | 2.9 ± 0.7** | 597.2 ± 33.2*** | 1.5 ± 0.1*** | -0.8 ± 0.1*** |
| Ol.bot.cau-Hybrid | Ol.cap.bal-Hybrid | -0.4 ± 0.2NS | -0.5 ± 0.3NS | -0.3 ± 0.2NS | 0.8 ± 0.2* | 0.6 ± 0.7NS | 242.5 ± 33.3*** | 1.1 ± 0.1*** | -0.7 ± 0.1*** |
| Ol.bot.cau-Hybrid | Ol.cap.bin-Inbred | 0.1 ± 0.2NS | -2.0 ± 0.3*** | 0.2 ± 0.2NS | 1.3 ± 0.2*** | -0.4 ± 0.7NS | 603.0 ± 33.0*** | 0.9 ± 0.1*** | -1.3 ± 0.1*** |
| Ol.bot.cau-Hybrid | Ol.cap.bin-Hybrid | 0.6 ± 0.2NS | -0.6 ± 0.3NS | 0.5 ± 0.2NS | 0.4 ± 0.2NS | -3.6 ± 0.7*** | 126.7 ± 33.1** | 0.7 ± 0.1*** | -1.0 ± 0.1*** |
| Ol.bot.cau-Hybrid | Ol.ita.pre-Inbred | -0.6 ± 0.2NS | -0.7 ± 0.3NS | 0.0 ± 0.2NS | 1.4 ± 0.2*** | 3.1 ± 0.7** | 509.8 ± 35.1*** | 1.0 ± 0.1*** | -0.7 ± 0.1*** |
| Ol.bot.cau-Hybrid | Ol.ita.pre-Hybrid | -0.2 ± 0.2NS | -1.0 ± 0.3NS | -0.2 ± 0.2NS | 1.2 ± 0.2*** | 0.4 ± 0.7NS | 268.1 ± 35.1*** | 1.0 ± 0.1*** | -0.7 ± 0.1*** |
| Ol.cap.bad-Inbred | Ol.cap.bad-Hybrid | 1.3 ± 0.2*** | 2.4 ± 0.4*** | 0.9 ± 0.2*** | -1.7 ± 0.2*** | -3.9 ± 0.8*** | -574.5 ± 37.1*** | -0.8 ± 0.2*** | 0.6 ± 0.1*** |
| Ol.cap.bad-Inbred | Ol.cap.bal-Inbred | -0.3 ± 0.2NS | 2.3 ± 0.3*** | -0.1 ± 0.2NS | -0.8 ± 0.2NS | 3.4 ± 0.7*** | -78.8 ± 35.0NS | -0.3 ± 0.1NS | 0.7 ± 0.1*** |
| Ol.cap.bad-Inbred | Ol.cap.bal-Hybrid | 0.9 ± 0.2** | 2.7 ± 0.3*** | 0.2 ± 0.2NS | -1.6 ± 0.2*** | 1.1 ± 0.7NS | -433.5 ± 35.1*** | -0.7 ± 0.1*** | 0.7 ± 0.1*** |
| Ol.cap.bad-Inbred | Ol.cap.bin-Inbred | 1.4 ± 0.2*** | 1.2 ± 0.3* | 0.7 ± 0.2** | -1.2 ± 0.2*** | 0.2 ± 0.7NS | -73.0 ± 34.9NS | -0.9 ± 0.1*** | 0.1 ± 0.1NS |
| Ol.cap.bad-Inbred | Ol.cap.bin-Hybrid | 1.8 ± 0.2*** | 2.6 ± 0.3*** | 1.1 ± 0.2*** | -2 ± 0.2*** | -3.1 ± 0.7** | -549.4 ± 34.9*** | -1.1 ± 0.1*** | 0.5 ± 0.1** |
| Ol.cap.bad-Inbred | Ol.ita.pre-Inbred | 0.7 ± 0.2NS | 2.5 ± 0.4*** | 0.6 ± 0.2NS | -1.1 ± 0.2*** | 3.6 ± 0.8*** | -166.2 ± 36.8*** | -0.8 ± 0.2*** | 0.8 ± 0.1*** |
| Ol.cap.bad-Inbred | Ol.ita.pre-Hybrid | 1.1 ± 0.2*** | 2.2 ± 0.4*** | 0.3 ± 0.2NS | -1.2 ± 0.2*** | 0.9 ± 0.8NS | -407.9 ± 36.8*** | -0.8 ± 0.2*** | 0.8 ± 0.1*** |
| Ol.cap.bad-Hybrid | Ol.cap.bal-Inbred | -1.5 ± 0.2*** | -0.1 ± 0.3NS | -1.0 ± 0.2*** | 1.0 ± 0.2** | 7.4 ± 0.7*** | 495.7 ± 35.0*** | 0.4 ± 0.1NS | 0.1 ± 0.1NS |
| Ol.cap.bad-Hybrid | Ol.cap.bal-Hybrid | -0.4 ± 0.2NS | 0.2 ± 0.3NS | -0.6 ± 0.2* | 0.1 ± 0.2NS | 5.0 ± 0.7*** | 140.9 ± 35.1** | 0.1 ± 0.1NS | 0.2 ± 0.1NS |
| Ol.cap.bad-Hybrid | Ol.cap.bin-Inbred | 0.1 ± 0.2NS | -1.3 ± 0.3* | -0.2 ± 0.2NS | 0.6 ± 0.2NS | 4.1 ± 0.7*** | 501.5 ± 34.9*** | -0.1 ± 0.1NS | -0.5 ± 0.1*** |
| Ol.cap.bad-Hybrid | Ol.cap.bin-Hybrid | 0.6 ± 0.2NS | 0.1 ± 0.3NS | 0.2 ± 0.2NS | -0.3 ± 0.2NS | 0.8 ± 0.7NS | 25.1 ± 34.9NS | -0.3 ± 0.1NS | -0.1 ± 0.1NS |
| Ol.cap.bad-Hybrid | Ol.ita.pre-Inbred | -0.6 ± 0.2NS | 0.1 ± 0.4NS | -0.3 ± 0.2NS | 0.7 ± 0.2NS | 7.6 ± 0.8*** | 408.2 ± 36.8*** | 0.0 ± 0.2NS | 0.2 ± 0.1NS |
| Ol.cap.bad-Hybrid | Ol.ita.pre-Hybrid | -0.2 ± 0.2NS | -0.2 ± 0.4NS | -0.6 ± 0.2NS | 0.5 ± 0.2NS | 4.9 ± 0.8*** | 166.6 ± 36.8*** | -0.1 ± 0.2NS | 0.2 ± 0.1NS |
| Ol.cap.bal-Inbred | Ol.cap.bal-Hybrid | 1.2 ± 0.2*** | 0.4 ± 0.3NS | 0.4 ± 0.2NS | -0.9 ± 0.2** | -2.3 ± 0.7* | -354.8 ± 32.8*** | -0.3 ± 0.1NS | 0.1 ± 0.1NS |
| Ol.cap.bal-Inbred | Ol.cap.bin-Inbred | 1.7 ± 0.2*** | -1.1 ± 0.3* | 0.8 ± 0.2*** | -0.4 ± 0.2NS | -3.3 ± 0.7*** | 5.8 ± 32.6NS | -0.5 ± 0.1** | -0.5 ± 0.1*** |
| Ol.cap.bal-Inbred | Ol.cap.bin-Hybrid | 2.1 ± 0.2*** | 0.3 ± 0.3NS | 1.2 ± 0.2*** | -1.2 ± 0.2*** | -6.5 ± 0.7*** | -470.6 ± 32.7*** | -0.8 ± 0.1*** | -0.2 ± 0.1NS |
| Ol.cap.bal-Inbred | Ol.ita.pre-Inbred | 0.9 ± 0.2*** | 0.2 ± 0.3NS | 0.7 ± 0.2* | -0.3 ± 0.2NS | 0.2 ± 0.7NS | -87.5 ± 34.7NS | -0.5 ± 0.1NS | 0.1 ± 0.1NS |
| Ol.cap.bal-Inbred | Ol.ita.pre-Hybrid | 1.3 ± 0.2*** | 0.0 ± 0.3NS | 0.5 ± 0.2NS | -0.5 ± 0.2NS | -2.5 ± 0.7* | -329.1 ± 34.7*** | -0.5 ± 0.1* | 0.1 ± 0.1NS |
| Ol.cap.bal-Hybrid | Ol.cap.bin-Inbred | 0.5 ± 0.2NS | -1.5 ± 0.3*** | 0.5 ± 0.2NS | 0.5 ± 0.2NS | -0.9 ± 0.7NS | 360.5 ± 32.7*** | -0.2 ± 0.1NS | -0.6 ± 0.1*** |
| Ol.cap.bal-Hybrid | Ol.cap.bin-Hybrid | 0.9 ± 0.2*** | -0.1 ± 0.3NS | 0.8 ± 0.2*** | -0.4 ± 0.2NS | -4.2 ± 0.7*** | -115.8 ± 32.7* | -0.4 ± 0.1NS | -0.3 ± 0.1NS |
| Ol.cap.bal-Hybrid | Ol.ita.pre-Inbred | -0.2 ± 0.2NS | -0.2 ± 0.3NS | 0.3 ± 0.2NS | 0.6 ± 0.2NS | 2.5 ± 0.7* | 267.3 ± 34.7*** | -0.1 ± 0.1NS | 0.0 ± 0.1NS |
| Ol.cap.bal-Hybrid | Ol.ita.pre-Hybrid | 0.2 ± 0.2NS | -0.4 ± 0.3NS | 0.1 ± 0.2NS | 0.4 ± 0.2NS | -0.2 ± 0.7NS | 25.6 ± 34.7NS | -0.2 ± 0.1NS | 0.0 ± 0.1NS |
| Ol.cap.bin-Inbred | Ol.cap.bin-Hybrid | 0.4 ± 0.2NS | 1.4 ± 0.3** | 0.4 ± 0.2NS | -0.8 ± 0.2** | -3.3 ± 0.7*** | -476.4 ± 32.5*** | -0.3 ± 0.1NS | 0.3 ± 0.1* |
| Ol.cap.bin-Inbred | Ol.ita.pre-Inbred | -0.7 ± 0.2* | 1.3 ± 0.3** | -0.2 ± 0.2NS | 0.1 ± 0.2NS | 3.5 ± 0.7*** | -93.2 ± 34.5NS | 0.0 ± 0.1NS | 0.6 ± 0.1*** |
| Ol.cap.bin-Inbred | Ol.ita.pre-Hybrid | -0.3 ± 0.2NS | 1.1 ± 0.3NS | -0.4 ± 0.2NS | -0.1 ± 0.2NS | 0.8 ± 0.7NS | -334.9 ± 34.5*** | 0.0 ± 0.1NS | 0.7 ± 0.1*** |
| Ol.cap.bin-Hybrid | Ol.ita.pre-Inbred | -1.2 ± 0.2*** | -0.1 ± 0.3NS | -0.5 ± 0.2NS | 0.9 ± 0.2** | 6.7 ± 0.7*** | 383.1 ± 34.5*** | 0.3 ± 0.1NS | 0.3 ± 0.1NS |
| Ol.cap.bin-Hybrid | Ol.ita.pre-Hybrid | -0.7 ± 0.2* | -0.3 ± 0.3NS | -0.7 ± 0.2** | 0.8 ± 0.2* | 4.0 ± 0.7*** | 141.5 ± 34.5** | 0.3 ± 0.1NS | 0.3 ± 0.1NS |
| Ol.ita.pre-Inbred | Ol.ita.pre-Hybrid | 0.4 ± 0.2NS | -0.3 ± 0.4NS | -0.2 ± 0.2NS | -0.2 ± 0.2NS | -2.7 ± 0.8* | -241.6 ± 36.5*** | 0.0 ± 0.2NS | 0.0 ± 0.1NS |
| Ol.alb.nrc-MPH | Ol.alb.nrc-NPH | -0.3 ± 0.3NS | -4.8 ± 0.9*** | -0.4 ± 0.1NS | 3.1 ± 0.4*** | 3.1 ± 0.4*** | 6.2 ± 1.2*** | 1.3 ± 0.2*** | -0.8 ± 0.3NS |
| Ol.alb.nrc-MPH | Ol.bot.cau-MPH | 0.3 ± 0.3NS | 0.1 ± 0.9NS | 0.1 ± 0.1NS | -0.1 ± 0.8NS | 0.8 ± 0.4NS | 0.4 ± 1.2NS | 0.1 ± 0.2NS | 0.0 ± 0.3NS |
| Ol.alb.nrc-MPH | Ol.bot.cau-NPH | 0.2 ± 0.3NS | -4.5 ± 0.9*** | -0.2 ± 0.1NS | 2.3 ± 0.8NS | 3.9 ± 0.4*** | 6.8 ± 1.2*** | 1.0 ± 0.2*** | -1.0 ± 0.3NS |
| Ol.alb.nrc-MPH | Ol.cap.bad-MPH | 0.1 ± 0.3NS | 3.0 ± 0.9NS | 0.4 ± 0.1NS | -1.0 ± 0.9NS | 0.4 ± 0.4NS | 0.8 ± 1.3NS | 0.0 ± 0.2NS | 0.1 ± 0.4NS |
| Ol.alb.nrc-MPH | Ol.cap.bad-NPH | -0.7 ± 0.3NS | -4.1 ± 0.9** | -0.2 ± 0.1NS | 4.3 ± 0.9*** | 2.6 ± 0.4*** | 8.2 ± 1.3*** | 1.8 ± 0.2*** | -2.3 ± 0.4*** |
| Ol.alb.nrc-MPH | Ol.cap.bal-MPH | -0.4 ± 0.3NS | -0.7 ± 0.9NS | 0.0 ± 0.1NS | 1.0 ± 0.8NS | 0.6 ± 0.4NS | 4.8 ± 1.2** | 0.3 ± 0.2NS | -0.4 ± 0.3NS |
| Ol.alb.nrc-MPH | Ol.cap.bal-NPH | -1.6 ± 0.3*** | -4.6 ± 0.9*** | -0.4 ± 0.1** | 4.8 ± 0.8*** | 2.0 ± 0.4*** | 9.6 ± 1.2*** | 1.4 ± 0.2*** | -1.2 ± 0.3* |
| Ol.alb.nrc-MPH | Ol.cap.bin-MPH | -0.1 ± 0.3NS | 1.1 ± 0.9NS | 0.2 ± 0.1NS | 0.5 ± 0.8NS | 1.1 ± 0.4NS | 2.8 ± 1.2NS | 0.3 ± 0.2NS | -0.6 ± 0.3NS |
| Ol.alb.nrc-MPH | Ol.cap.bin-NPH | 0.5 ± 0.3NS | -4.3 ± 0.9*** | 0.0 ± 0.1NS | 3.6 ± 0.8*** | 3.5 ± 0.4*** | 9.4 ± 1.2*** | 1.2 ± 0.2*** | -2.8 ± 0.3*** |
| Ol.alb.nrc-MPH | Ol.ita.pre-MPH | -1.0 ± 0.3NS | -2.6 ± 0.9NS | -0.3 ± 0.1NS | 3.2 ± 0.9* | 0.7 ± 0.4NS | 7.1 ± 1.3*** | 0.5 ± 0.2NS | -0.4 ± 0.3NS |
| Comparisons | | Days to flowering | Duration of flowering (day) | Days to maturity | Grain-filling period (day) | Plant height (cm) | Seed yield (kg ha^-1^) | Seed oil (%) | Seed protein (%) |
| Ol.alb.nrc-MPH | Ol.ita.pre-NPH | -1.3 ± 0.3** | -5.9 ± 0.9*** | -0.4 ± 0.1* | 6.4 ± 0.9*** | 2.8 ± 0.4*** | 10.9 ± 1.3*** | 1.1 ± 0.2*** | -1.1 ± 0.3NS |
| Ol.alb.nrc-NPH | Ol.bot.cau-MPH | 0.7 ± 0.3NS | 4.9 ± 0.9*** | 0.5 ± 0.1*** | -2.6 ± 0.8NS | -2.2 ± 0.4*** | -5.9 ± 1.2*** | -1.3 ± 0.2*** | 0.7 ± 0.3NS |
| Ol.alb.nrc-NPH | Ol.bot.cau-NPH | 0.6 ± 0.3NS | 0.3 ± 0.9NS | 0.2 ± 0.1NS | -0.2 ± 0.8NS | 0.8 ± 0.4NS | 0.5 ± 1.2NS | -0.4 ± 0.2NS | -0.2 ± 0.3NS |
| Ol.alb.nrc-NPH | Ol.cap.bad-MPH | 0.5 ± 0.3NS | 7.8 ± 0.9*** | 0.7 ± 0.1*** | -3.5 ± 0.9** | -2.7 ± 0.4*** | -5.4 ± 1.3** | -1.3 ± 0.2*** | 0.9 ± 0.4NS |
| Ol.alb.nrc-NPH | Ol.cap.bad-NPH | -0.4 ± 0.3NS | 0.7 ± 0.9NS | 0.2 ± 0.1NS | 1.9 ± 0.9NS | -0.5 ± 0.4NS | 1.9 ± 1.3NS | 0.5 ± 0.2NS | -1.5 ± 0.4** |
| Ol.alb.nrc-NPH | Ol.cap.bal-MPH | -0.1 ± 0.3NS | 4.1 ± 0.9*** | 0.3 ± 0.1NS | -1.4 ± 0.8NS | -2.5 ± 0.4*** | -1.5 ± 1.2NS | -1.1 ± 0.2*** | 0.4 ± 0.3NS |
| Ol.alb.nrc-NPH | Ol.cap.bal-NPH | -1.2 ± 0.3** | 0.3 ± 0.9NS | -0.1 ± 0.1NS | 2.4 ± 0.8NS | -1.0 ± 0.4NS | 3.3 ± 1.2NS | 0.0 ± 0.2NS | -0.4 ± 0.3NS |
| Ol.alb.nrc-NPH | Ol.cap.bin-MPH | 0.3 ± 0.3NS | 5.9 ± 0.9*** | 0.6 ± 0.1*** | -2.0 ± 0.8NS | -2.0 ± 0.4*** | -3.4 ± 1.2NS | -1.1 ± 0.2*** | 0.2 ± 0.3NS |
| Ol.alb.nrc-NPH | Ol.cap.bin-NPH | 0.8 ± 0.3NS | 0.5 ± 0.9NS | 0.3 ± 0.1NS | 1.2 ± 0.8NS | 0.5 ± 0.4NS | 3.2 ± 1.2NS | -0.1 ± 0.2NS | -2.0 ± 0.3*** |
| Ol.alb.nrc-NPH | Ol.ita.pre-MPH | -0.7 ± 0.3NS | 2.3 ± 0.9NS | 0.1 ± 0.1NS | 0.7 ± 0.9NS | -2.3 ± 0.4*** | 0.8 ± 1.3NS | -0.9 ± 0.2** | 0.4 ± 0.3NS |
| Ol.alb.nrc-NPH | Ol.ita.pre-NPH | -1.0 ± 0.3NS | -1.1 ± 0.9NS | 0.0 ± 0.1NS | 3.9 ± 0.9*** | -0.3 ± 0.4NS | 4.6 ± 1.3* | -0.2 ± 0.2NS | -0.3 ± 0.3NS |
| Ol.bot.cau-MPH | Ol.bot.cau-NPH | -0.1 ± 0.3NS | -4.6 ± 0.9*** | -0.3 ± 0.1NS | 2.4 ± 0.8NS | 3.0 ± 0.4*** | 6.4 ± 1.2*** | 0.9 ± 0.2*** | -0.9 ± 0.3NS |
| Ol.bot.cau-MPH | Ol.cap.bad-MPH | -0.2 ± 0.3NS | 2.9 ± 0.9NS | 0.2 ± 0.1NS | -0.9 ± 0.9NS | -0.5 ± 0.4NS | 0.4 ± 1.2NS | 0.0 ± 0.2NS | 0.1 ± 0.3NS |
| Ol.bot.cau-MPH | Ol.cap.bad-NPH | -1.1 ± 0.3* | -4.2 ± 0.9*** | -0.3 ± 0.1NS | 4.5 ± 0.9*** | 1.8 ± 0.4*** | 7.8 ± 1.2*** | 1.7 ± 0.2*** | -2.3 ± 0.3*** |
| Ol.bot.cau-MPH | Ol.cap.bal-MPH | -0.7 ± 0.3NS | -0.8 ± 0.9NS | -0.1 ± 0.1NS | 1.2 ± 0.8NS | -0.3 ± 0.4NS | 4.4 ± 1.2** | 0.2 ± 0.2NS | -0.3 ± 0.3NS |
| Ol.bot.cau-MPH | Ol.cap.bal-NPH | -1.9 ± 0.3*** | -4.7 ± 0.9*** | -0.6 ± 0.1*** | 4.9 ± 0.8*** | 1.2 ± 0.4NS | 9.2 ± 1.2*** | 1.3 ± 0.2*** | -1.2 ± 0.3* |
| Ol.bot.cau-MPH | Ol.cap.bin-MPH | -0.4 ± 0.3NS | 1.0 ± 0.9NS | 0.1 ± 0.1NS | 0.6 ± 0.8NS | 0.2 ± 0.4NS | 2.5 ± 1.2NS | 0.2 ± 0.2NS | -0.5 ± 0.3NS |
| Ol.bot.cau-MPH | Ol.cap.bin-NPH | 0.2 ± 0.3NS | -4.4 ± 0.9*** | -0.1 ± 0.1NS | 3.7 ± 0.8*** | 2.7 ± 0.4*** | 9.1 ± 1.2*** | 1.1 ± 0.2*** | -2.7 ± 0.3*** |
| Ol.bot.cau-MPH | Ol.ita.pre-MPH | -1.4 ± 0.3*** | -2.7 ± 0.9NS | -0.4 ± 0.1* | 3.3 ± 0.9** | -0.1 ± 0.4NS | 6.7 ± 1.2*** | 0.4 ± 0.2NS | -0.4 ± 0.3NS |
| Ol.bot.cau-MPH | Ol.ita.pre-NPH | -1.6 ± 0.3*** | -6.0 ± 0.9*** | -0.5 ± 0.1*** | 6.5 ± 0.9*** | 1.9 ± 0.4*** | 10.5 ± 1.2*** | 1.0 ± 0.2*** | -1.0 ± 0.3NS |
| Ol.bot.cau-NPH | Ol.cap.bad-MPH | -0.1 ± 0.3NS | 7.5 ± 0.9*** | 0.5 ± 0.1*** | -3.3 ± 0.9** | -3.5 ± 0.4*** | -6.0 ± 1.2*** | -0.9 ± 0.2*** | 1.1 ± 0.3NS |
| Ol.bot.cau-NPH | Ol.cap.bad-NPH | -1.0 ± 0.3NS | 0.4 ± 0.9NS | 0.0 ± 0.1NS | 2.1 ± 0.9NS | -1.3 ± 0.4NS | 1.4 ± 1.2NS | 0.8 ± 0.2** | -1.3 ± 0.3** |
| Ol.bot.cau-NPH | Ol.cap.bal-MPH | -0.6 ± 0.3NS | 3.8 ± 0.9** | 0.1 ± 0.1NS | -1.2 ± 0.8NS | -3.3 ± 0.4*** | -2.0 ± 1.2NS | -0.7 ± 0.2* | 0.6 ± 0.3NS |
| Ol.bot.cau-NPH | Ol.cap.bal-NPH | -1.8 ± 0.3*** | -0.1 ± 0.9NS | -0.3 ± 0.1NS | 2.5 ± 0.8NS | -1.8 ± 0.4*** | 2.8 ± 1.2NS | 0.4 ± 0.2NS | -0.2 ± 0.3NS |
| Ol.bot.cau-NPH | Ol.cap.bin-MPH | -0.3 ± 0.3NS | 5.6 ± 0.9*** | 0.4 ± 0.1* | -1.8 ± 0.8NS | -2.8 ± 0.4*** | -3.9 ± 1.2* | -0.7 ± 0.2* | 0.4 ± 0.3NS |
| Ol.bot.cau-NPH | Ol.cap.bin-NPH | 0.3 ± 0.3NS | 0.2 ± 0.9NS | 0.1 ± 0.1NS | 1.3 ± 0.8NS | -0.3 ± 0.4NS | 2.7 ± 1.2NS | 0.2 ± 0.2NS | -1.8 ± 0.3*** |
| Ol.bot.cau-NPH | Ol.ita.pre-MPH | -1.3 ± 0.3** | 1.9 ± 0.9NS | -0.1 ± 0.1NS | 0.9 ± 0.9NS | -3.1 ± 0.4*** | 0.3 ± 1.2NS | -0.5 ± 0.2NS | 0.6 ± 0.3NS |
| Ol.bot.cau-NPH | Ol.ita.pre-NPH | -1.5 ± 0.3*** | -1.4 ± 0.9NS | -0.2 ± 0.1NS | 4.1 ± 0.9*** | -1.1 ± 0.4NS | 4.1 ± 1.2* | 0.1 ± 0.2NS | -0.1 ± 0.3NS |
| Ol.cap.bad-MPH | Ol.cap.bad-NPH | -0.9 ± 0.3NS | -7.1 ± 1.0*** | -0.6 ± 0.1*** | 5.4 ± 0.9*** | 2.2 ± 0.4*** | 7.4 ± 1.3*** | 1.8 ± 0.2*** | -2.4 ± 0.4*** |
| Ol.cap.bad-MPH | Ol.cap.bal-MPH | -0.5 ± 0.3NS | -3.7 ± 0.9** | -0.4 ± 0.1* | 2.1 ± 0.8NS | 0.2 ± 0.4NS | 4.0 ± 1.2NS | 0.2 ± 0.2NS | -0.5 ± 0.3NS |
| Ol.cap.bad-MPH | Ol.cap.bal-NPH | -1.7 ± 0.3*** | -7.6 ± 0.9*** | -0.8 ± 0.1*** | 5.9 ± 0.8*** | 1.7 ± 0.4** | 8.8 ± 1.2*** | 1.3 ± 0.2*** | -1.3 ± 0.3** |
| Ol.cap.bad-MPH | Ol.cap.bin-MPH | -0.2 ± 0.3NS | -1.9 ± 0.9NS | -0.1 ± 0.1NS | 1.5 ± 0.8NS | 0.7 ± 0.4NS | 2.0 ± 1.2NS | 0.2 ± 0.2NS | -0.7 ± 0.3NS |
| Ol.cap.bad-MPH | Ol.cap.bin-NPH | 0.4 ± 0.3NS | -7.3 ± 0.9*** | -0.4 ± 0.1* | 4.7 ± 0.8*** | 3.2 ± 0.4*** | 8.6 ± 1.2*** | 1.2 ± 0.2*** | -2.9 ± 0.3*** |
| Ol.cap.bad-MPH | Ol.ita.pre-MPH | -1.2 ± 0.3* | -5.6 ± 1.0*** | -0.6 ± 0.1*** | 4.2 ± 0.9*** | 0.4 ± 0.4NS | 6.3 ± 1.3*** | 0.4 ± 0.2NS | -0.5 ± 0.4NS |
| Ol.cap.bad-MPH | Ol.ita.pre-NPH | -1.4 ± 0.3*** | -8.9 ± 1.0*** | -0.7 ± 0.1*** | 7.5 ± 0.9*** | 2.4 ± 0.4*** | 10.1 ± 1.3*** | 1.1 ± 0.2*** | -1.2 ± 0.4NS |
| Ol.cap.bad-NPH | Ol.cap.bal-MPH | 0.3 ± 0.3NS | 3.4 ± 0.9* | 0.2 ± 0.1NS | -3.3 ± 0.8** | -2.0 ± 0.4*** | -3.4 ± 1.2NS | -1.5 ± 0.2*** | 1.9 ± 0.3*** |
| Ol.cap.bad-NPH | Ol.cap.bal-NPH | -0.8 ± 0.3NS | -0.5 ± 0.9NS | -0.2 ± 0.1NS | 0.5 ± 0.8NS | -0.6 ± 0.4NS | 1.4 ± 1.2NS | -0.5 ± 0.2NS | 1.1 ± 0.3NS |
| Ol.cap.bad-NPH | Ol.cap.bin-MPH | 0.7 ± 0.3NS | 5.2 ± 0.9*** | 0.4 ± 0.1** | -3.9 ± 0.8*** | -1.5 ± 0.4** | -5.4 ± 1.2*** | -1.5 ± 0.2*** | 1.7 ± 0.3*** |
| Ol.cap.bad-NPH | Ol.cap.bin-NPH | 1.2 ± 0.3** | -0.2 ± 0.9NS | 0.2 ± 0.1NS | -0.7 ± 0.8NS | 0.9 ± 0.4NS | 1.3 ± 1.2NS | -0.6 ± 0.2NS | -0.5 ± 0.3NS |
| Ol.cap.bad-NPH | Ol.ita.pre-MPH | -0.3 ± 0.3NS | 1.5 ± 1.0NS | -0.1 ± 0.1NS | -1.1 ± 0.9NS | -1.9 ± 0.4*** | -1.1 ± 1.3NS | -1.4 ± 0.2*** | 1.9 ± 0.4*** |
| Ol.cap.bad-NPH | Ol.ita.pre-NPH | -0.6 ± 0.3NS | -1.8 ± .01NS | -0.2 ± 0.1NS | 2.1 ± 0.9NS | 0.2 ± 0.4NS | 2.7 ± 1.3NS | -0.7 ± 0.2NS | 1.2 ± 0.4* |
| Ol.cap.bal-MPH | Ol.cap.bal-NPH | -1.2 ± 0.3** | -3.9 ± 0.9*** | -0.4 ± 0.1** | 3.8 ± 0.8*** | 1.5 ± 0.4** | 4.8 ± 1.2** | 1.1 ± 0.2*** | -0.9 ± 0.3NS |
| Ol.cap.bal-MPH | Ol.cap.bin-MPH | 0.3 ± 0.3NS | 1.8 ± 0.9NS | 0.2 ± 0.1NS | -0.6 ± 0.8NS | 0.5 ± 0.4NS | -2.0 ± 1.1NS | 0.0 ± 0.2NS | -0.2 ± 0.3NS |
| Ol.cap.bal-MPH | Ol.cap.bin-NPH | 0.9 ± 0.3NS | -3.6 ± 0.9** | 0.0 ± 0.1NS | 2.6 ± 0.8NS | 3.0 ± 0.4*** | 4.7 ± 1.1** | 0.9 ± 0.2*** | -2.4 ± 0.3*** |
| Ol.cap.bal-MPH | Ol.ita.pre-MPH | -0.6 ± 0.3NS | -1.9 ± 0.9NS | -0.3 ± 0.1NS | 2.2 ± 0.8NS | 0.2 ± 0.4NS | 2.3 ± 1.2NS | 0.2 ± 0.2NS | -0.1 ± 0.3NS |
| Ol.cap.bal-MPH | Ol.ita.pre-NPH | -0.9 ± 0.3NS | -5.2 ± 0.9*** | -0.4 ± 0.1* | 5.4 ± 0.8*** | 2.2 ± 0.4*** | 6.1 ± 1.2*** | 0.8 ± 0.2** | -0.7 ± 0.3NS |
| Comparisons | | Days to flowering | Duration of flowering (day) | Days to maturity | Grain-filling period (day) | Plant height (cm) | Seed yield (kg ha^-1^) | Seed oil (%) | Seed protein (%) |
| Ol.cap.bal-NPH | Ol.cap.bin-MPH | 1.5 ± 0.3*** | 5.7 ± 0.9*** | 0.7 ± 0.1*** | -4.4 ± 0.8*** | -1.0 ± 0.4NS | -6.8 ± 1.1*** | -1.1 ± 0.2*** | 0.6 ± 0.3NS |
| Ol.cap.bal-NPH | Ol.cap.bin-NPH | 2.1 ± 0.3*** | 0.2 ± 0.9NS | 0.4 ± 0.1** | -1.2 ± 0.8NS | 1.5 ± 0.4** | -0.1 ± 1.1NS | -0.1 ± 0.2NS | -1.6 ± 0.3*** |
| Ol.cap.bal-NPH | Ol.ita.pre-MPH | 0.6 ± 0.3NS | 2.0 ± 0.9NS | 0.2 ± 0.1NS | -1.6 ± 0.8NS | -1.3 ± 0.4* | -2.5 ± 1.2NS | -0.9 ± 0.2*** | 0.8 ± 0.3NS |
| Ol.cap.bal-NPH | Ol.ita.pre-NPH | 0.3 ± 0.3NS | -1.4 ± 0.9NS | 0.1 ± 0.1NS | 1.6 ± 0.8NS | 0.7 ± 0.4NS | 1.3 ± 1.2NS | -0.3 ± 0.2NS | 0.1 ± 0.3NS |
| Ol.cap.bin-MPH | Ol.cap.bin-NPH | 0.6 ± 0.3NS | -5.4 ± 0.8*** | -0.2 ± 0.1NS | 3.2 ± 0.8** | 2.5 ± 0.4*** | 6.6 ± 1.1*** | 0.9 ± 0.2*** | -2.2 ± 0.3*** |
| Ol.cap.bin-MPH | Ol.ita.pre-MPH | -1.0 ± 0.3NS | -3.7 ± 0.9** | -0.5 ± 0.1*** | 2.8 ± 0.8NS | -0.3 ± 0.4NS | 4.3 ± 1.2* | 0.2 ± 0.2NS | 0.2 ± 0.3NS |
| Ol.cap.bin-MPH | Ol.ita.pre-NPH | -1.2 ± 0.3** | -7.0 ± 0.9*** | -0.6 ± 0.1*** | 6.0 ± 0.8*** | 1.7 ± 0.4*** | 8.1 ± 1.2*** | 0.8 ± 0.2** | -0.5 ± 0.3NS |
| Ol.cap.bin-NPH | Ol.ita.pre-MPH | -1.5 ± 0.3*** | 1.8 ± 0.9NS | -0.2 ± 0.1NS | -0.4 ± 0.8NS | -2.8 ± 0.4*** | -2.4 ± 1.2NS | -0.8 ± 0.2* | 2.4 ± 0.3*** |
| Ol.cap.bin-NPH | Ol.ita.pre-NPH | -1.8 ± 0.3*** | -1.6 ± 0.9NS | -0.4 ± 0.1* | 2.8 ± 0.8* | -0.8 ± 0.4NS | 1.4 ± 1.2NS | -0.1 ± 0.2NS | 1.7 ± 0.3*** |
| Ol.ita.pre-MPH | Ol.ita.pre-NPH | -0.3 ± 0.3NS | -3.4 ± 1.0* | -0.1 ± 0.1NS | 3.2 ± 0.9* | 2.0 ± 0.4*** | 3.8 ± 1.3NS | 0.6 ± 0.2NS | -0.7 ± 0.4NS |

^*^ Significant at P < 0.05, ^**^ Significant at P < 0.01, ^***^ Significant at P < 0.001, ^NS^ Not significant

^1^ Ol.alb.nrc = *B. napus* (A04-73NA) × *B. oleracea* var. *alboglabra* line NRC-PBI; Ol.bot.cau = *B. napus* (A04-73NA) × *B. oleracea* var. *botrytis* cv. BARI cauliflower; Ol.cap.bad = *B. napus* (A04-73NA) × *B. oleracea* var. *capitata* cv. Badger Shipper; Ol.cap.bin = *B. napus* (A04-73NA) × *B.* *oleracea* var*. capitata* cv. Bindsachsener; Ol.cap.bal = *B. napus* (A04-73NA) × *B.* *oleracea* var. *capitata* cv. Balbro; Ol.ita.pre = A04-73NA × *B. oleracea* var. *italica* cv. Premium Crop; Inbred = inbred line population; Hybrid = test hybrid population; MPH = mid-parent heterosis; NPH = heterosis over common *B. napus* parent

**Supplementary Table 5.** Comparison of the least square mean (± SE) values of the F_2_- and BC_1_-derived inbred lines, their test-hybrids, mid-parent heterosis (MPH), and heterosis over the common *B. napus* parent (NPH) for different agronomic and seed quality traits

| Comparisons | | Days to flowering | Duration of flowering (day) | Days to maturity | Grain-filling period (day) | Plant height (cm) | Seed yield (kg ha^-1^) | Seed oil (%) | Seed protein (%) |
| --- | --- | --- | --- | --- | --- | --- | --- | --- | --- |
| BC-Hybrid | BC-Inbred | -0.8 ± 0.1*** | -0.9 ± 0.2*** | -0.3 ± 0.1NS | 0.8 ± 0.1*** | 3.9 ± 0.4*** | 471.0 ± 21.3*** | 0.4 ± 0.1*** | -0.2 ± 0.1* |
| BC-Hybrid | F-Inbred | -1.4 ± 0.1*** | -1.3 ± 0.2*** | -0.6 ± 0.1*** | 1.2 ± 0.1*** | 2.5 ± 0.5*** | 469.4 ± 21.7*** | 0.3 ± 0.1** | -0.3 ± 0.1** |
| BC-Hybrid | F-Hybrid | -0.4 ± 0.1** | -0.4 ± 0.2NS | -0.2 ± 0.1NS | 0.4 ± 0.1* | -0.6 ± 0.5NS | 33.4 ± 21.7NS | 0.0 ± 0.1NS | -0.1 ± 0.1NS |
| BC-Inbred | F-Inbred | -0.5 ± 0.1*** | -0.4 ± 0.2NS | -0.3 ± 0.1* | 0.4 ± 0.1* | -1.4 ± 0.5* | -1.5 ± 21.7NS | -0.1 ± 0.1NS | 0.0 ± 0.1NS |
| BC-Inbred | F-Hybrid | 0.4 ± 0.1* | 0.5 ± 0.2* | 0.1 ± 0.1NS | -0.4 ± 0.1* | -4.5 ± 0.5*** | -437.5 ± 21.7*** | -0.4 ± 0.1*** | 0.2 ± 0.1NS |
| F-Inbred | F-Hybrid | -0.9 ± 0.1*** | -1.0 ± 0.2*** | -0.4 ± 0.1** | 0.8 ± 0.1*** | 3.1 ± 0.5*** | 436.0 ± 22.1*** | 0.3 ± 0.1** | -0.2 ± 0.1* |
| BC-NPH | BC-MPH | 0.0 ± 0.2NS | -4.5 ± 0.5*** | -0.3 ± 0.1*** | 3.0 ± 0.5*** | 2.7 ± 0.2*** | 6.2 ± 0.7*** | 1.2 ± 0.1*** | -1.3 ± 0.2*** |
| BC-NPH | F-MPH | -0.3 ± 0.2NS | 4.0 ± 0.5*** | 0.2 ± 0.1** | -2.2 ± 0.5*** | -2.5 ± 0.2*** | -5.3 ± 0.7*** | -1.2 ± 0.1*** | 1.4 ± 0.2*** |
| BC-NPH | F-NPH | -1.0 ± 0.2*** | -1.2 ± 0.5NS | -0.2 ± 0.1* | 1.5 ± 0.5** | -0.5 ± 0.2NS | 0.2 ± 0.7NS | -0.3 ± 0.1NS | 0.1 ± 0.2NS |
| BC-MPH | F-MPH | -0.4 ± 0.2NS | -0.4 ± 0.5NS | 0.0 ± 0.1NS | 0.8 ± 0.5NS | 0.1 ± 0.2NS | 0.8 ± 0.7NS | -0.1 ± 0.1NS | 0.0 ± 0.2NS |
| BC-MPH | F-NPH | -1.0 ± 0.2*** | -5.6 ± 0.5*** | -0.4 ± 0.1*** | 4.5 ± 0.5*** | 2.2 ± 0.2*** | 6.4 ± 0.7*** | 0.9 ± 0.1*** | -1.2 ± 0.2*** |
| F-MPH | F-NPH | -0.7 ± 0.2** | -5.2 ± 0.5*** | -0.4 ± 0.1*** | 3.7 ± 0.5*** | 2.1 ± 0.2*** | 5.5 ± 0.8*** | 1.0 ± 0.1*** | -1.3 ± 0.2*** |

^*^ Significant at P < 0.05, ^**^ Significant at P < 0.01, ^***^ Significant at P < 0.001, ^NS^ Not significant

Note: F-inbred = Inbred lines derived from F_2_; BC-inbred = Inbred lines derived from BC_1_; F-hybrid = Hybrids developed based on F_2_-derived inbred lines; BC-hybrid = Hybrids developed based on BC_1_-derived inbred lines; F-MPH: mid-parent heterosis for population derived from F_2_; BC-MPH: mid-parent heterosis for population derived from BC_1_; F-NPH: heterosis over the common *B. napus* parent for the population derived from F_2_; BC-NPH: heterosis over the common *B. napus* parent for the population derived from BC_1_

**Supplementary Table 6** Scores for different agronomic and seed quality traits after Principal Component Analysis (PCA) for the first three components

| Traits | PC1 | PC2 | PC3 |
| --- | --- | --- | --- |
| Test hybrids: |  |  |  |
| Days to flowering = DTF | -0.8217 | 1.7976 | -0.0992 |
| Duration of flowering = DOF | -1.8268 | 0.5785 | -0.09448 |
| Days to maturity = DTM | -0.8524 | 1.7437 | 0.41602 |
| Grain-filling period = DOGF | 1.8246 | -0.6553 | -0.16712 |
| Plant height = PH | -0.5313 | 0.2309 | -1.96986 |
| Seed yield = SY | 1.2934 | 0.597 | -1.0654 |
| Seed oil content = SOC | 1.5302 | 1.034 | -0.54861 |
| Seed protein content = SPC | -1.5214 | -1.2921 | -0.21204 |
| Mid-parent heterosis (MPH): |  |  |  |
| Days to flowering = DTF | 1.4242 | -0.6382 | 0.01531 |
| Duration of flowering = DOF | 1.6349 | -1.0947 | -0.09407 |
| Days to maturity = DTM | 1.4573 | -0.7593 | 0.39634 |
| Grain-filling period = DOGF | -1.5248 | 0.9603 | 0.37388 |
| Plant height = PH | -0.1356 | -0.7594 | 1.88871 |
| Seed yield = SY | -1.3601 | -0.25 | 0.80121 |
| Seed oil content = SOC | -1.5416 | -1.2738 | -0.49495 |
| Seed protein content = SPC | 1.3412 | 1.4029 | 0.66308 |

**
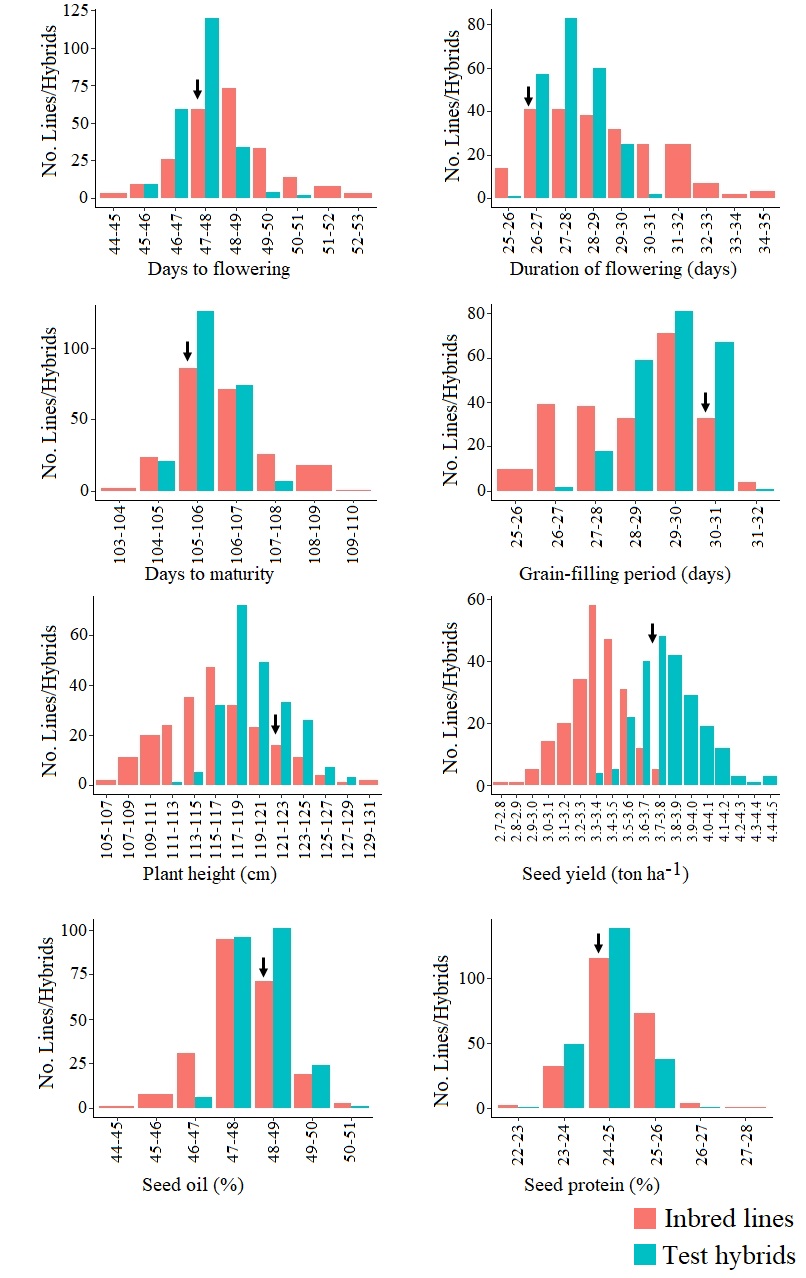
**

**Supplementary Figure 1.** Bar plots of the distribution of the inbred lines derived from six *Brassica napus* × *B. oleracea* interspecific crosses and their test hybrids for different agronomic and seed quality traits. Vertical arrows indicate the values of the *B. napus* parent. Blush colour bars represent inbred lines and teal bars represent the test hybrids.

**
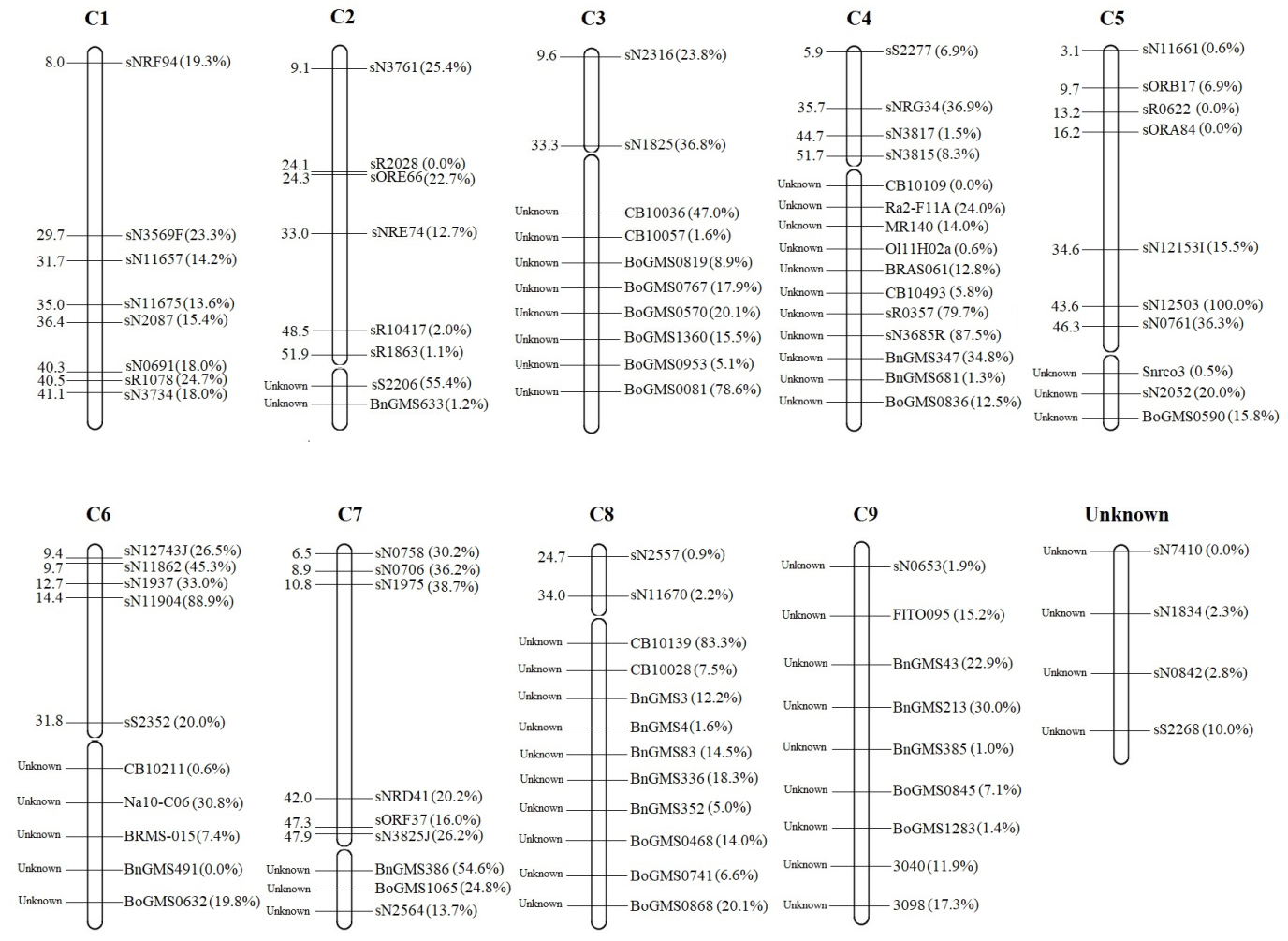
**

**Supplementary Figure 2.** Distribution of the 95 SSR markers used in the present study on different chromosomes of the C genome of *Brassica napus*. Map position of the markers showed on the left side of the chromosomes are in million bp. Markers from the C genome chromosomes which could not be positioned in *Brassica napus* (Chalhoub et al. 2014) or in *B. oleracea* (Parkin et al. 2014) reference genome are indicated by ‘unknown’ and drawn as a separate segment of the chromosome. The extent of heterozygosity of a marker in the test hybrid population deduced based on marker genotype of the 227 inbred lines derived from six *Brassica napus* × *B. oleracea* interspecific crosses and the common *B. napus* parent A04-73NA are shown in brackets after marker name.
